# Entomopathogenic *Metarhizium* and *Beauveria* species are associated with distinct geographic, management, and environmental factors, as well as plant communities

**DOI:** 10.1128/aem.02513-25

**Published:** 2026-07-21

**Authors:** Noemi Küng, Aaron Fox, Andreas Lüscher, Franco Widmer, Jürg Enkerli

**Affiliations:** 1 Molecular Ecology, Agroscope 419060, Zürich, Switzerland; 2 Plant Microbe Interaction, University of Basel 27209, Basel, Switzerland; 3 Environment Research Centre, Teagasc 159201 , Johnstown Castle, County Wexford, Ireland; 4 Forage Production and Grassland Systems, Agroscope 419060, Zürich, Switzerland; Royal Botanic Gardens Kew, Surrey, United Kingdom

**Keywords:** *Metarhizium*, *Beauveria*, amplicon sequencing, permanent grassland, ecological niche, plant communities, Landolt indicator values

## Abstract

Entomopathogenic fungi of the genera *Metarhizium* and *Beauveria* are important natural regulators of arthropod populations. To investigate their distribution and ecological drivers in real-world permanent grasslands, we did a survey analyzing fungal communities across 72 sites in Switzerland using ITS2 amplicon sequencing. We identified 10 *Metarhizium* and six *Beauveria* amplicon sequence variants (ASVs), with at least one ASV present at every site. Frequent ASVs included *M. robertsii*/*M. pingshaense*, *M. brunneum*, *B. bassiana*, and *B. brongniartii*. These ASVs showed distinct presence/absence and abundance patterns, indicating adaptation to climatic, soil physicochemical, and management-related factors. Factors such as soil pH, available phosphorus, temperature, bulk soil density, and management intensity significantly correlated with ASV distributions. For example, *M. robertsii*/*M. pingshaense* was widely distributed but strongly associated with soil pH, while *B. brongniartii* was restricted to subalpine regions, correlating with lower temperatures and nutrient levels. Plant communities, plant community-derived ecological indicator values (EIVs) reflecting environmental conditions, and indicator plant species analysis showed correlations with specific ASVs. On one hand, this suggests shared ecological niches and/or indicates possible direct plant–fungal interactions, consistent with the known endophytic or root-associated lifestyles of some entomopathogenic fungi. On the other hand, it suggests that plant-derived EIVs and individual indicator plant species may serve as effective indices for identifying habitats suitable for the presence and establishment of these entomopathogenic fungi. This approach may facilitate the evaluation of the suitability of particular environments for the application of *Metarhizium* and *Beauveria* strains as biological control agents.

**IMPORTANCE:**

The globally occurring fungal entomopathogens *Metarhizium* and *Beauveria* are key natural regulators of soil-dwelling arthropods and are widely used as biological control agents. Yet predicting their presence or potential to establish in particular habitats and provide their ecosystem function remains challenging. This survey study in real-world grasslands reveals distinct ecological niches characterized by climatic, soil physicochemical, and management parameters for different *Metarhizium* and *Beauveria* taxa. In addition, we demonstrate that plant community composition, plant-derived ecological indicator values, and individual indicator plant species are associated with *Metarhizium* and *Beauveria* presence and abundance. Our findings suggest that, within this regional context, vegetation data may serve as praxis-oriented proxies for efficient and non-invasive assessment of site suitability both for native fungal populations and biological control agents of selected *Metarhizium* and *Beauveria* taxa in grassland ecosystems.

## INTRODUCTION

The entomopathogenic fungal genera *Metarhizium* and *Beauveria* provide an important ecological function as natural regulators of arthropod populations. Their ability to actively infect and control arthropods and to remain and persist as natural components in the environment provides them with significant potential for use in biological pest control (1, 2). Such approaches, combined with integrated pest management (IPM) strategies, are promising safe alternatives to chemical pesticides, particularly in the context of the growing concerns over environmental and health impacts of synthetic insecticides. Next to their interaction with various arthropods, *Metarhizium* spp. and *Beauveria* spp. grow as saprophytes, and many of them are also known to interact with plants as rhizosphere colonizers and endophytes, and to be capable of switching between these lifestyles (1–5).

Biological control agents (BCAs), which are based on *Metarhizium* spp. and *Beauveria* spp., are commercialized worldwide (6). These products can be applied above ground, e.g., spray application on plants, as well as below ground by introducing products into the soil using, for instance, sowing machinery. Based on the host specificity of the fungus or fungal strain included in the BCA, products allow targeting multiple or single insect species in the case of fungi with a narrow host range. For instance, *M. brunneum* isolates, which generally have a wide host range, and *B. brongniartii*, with a narrow host range, are applied in grasslands (meadows, pastures, and lawns) to control white grubs, the soil-dwelling larvae of scarabaeoid beetles in Europe. While *M. brunneum* is used to control grubs of different species such as *Amphimallon solstitiale* or *Phyllopertha horticola* (7), *B. brongniartii* is used to specifically control white grubs of *Melolontha melolontha* (8). While the efficacy of fungal strains to infect and control a target insect is a major focus when developing new BCAs, it is well recognized that the environmental competence of a strain is also relevant and needs to be considered (9). A BCA must be adapted to prevailing environmental conditions in order to successfully establish and control the targeted pest insect. It is therefore of great importance to also know and recognize the characteristics of favorable environmental conditions for these fungi.

Soil is a dynamic and complex environment. Understanding the abiotic soil factors that favor the growth and activity of *Metarhizium* and *Beauveria* in this environment is crucial not only for improving their use in pest control strategies but also for protecting and supporting native populations and their function as natural antagonists. Climatic factors such as solar radiation, temperature, and humidity are known to affect infection rates and persistence (9, 10). While solar radiation is an important factor, as it may inactivate the fungus or its propagules on exposed surfaces (10, 11), its impact in a light-protected soil environment is likely minimal. In contrast, temperature and humidity are major limiting factors for the germination and growth of *Beauveria* and *Metarhizium* (10, 12–14), with optimal growth conditions varying by strain origin and environmental adaptation (15). The presence and abundance, as well as the community structure of *Beauveria* and *Metarhizium*, have been shown to also be affected by altitude, management practices, or land use (16–18). Furthermore, soil physicochemical characteristics, such as pH, soil texture, density and respiration, organic matter, and nutrient availability, play important roles in the presence of these fungi (16–18). *In vitro* studies have even suggested that not only presence but also virulence may be influenced by environmental factors. For example, pH has been shown to affect the expression of virulence factors such as cuticle-degrading enzymes and hydrophobins, potentially changing the ability of the fungus to penetrate the host insect cuticle (19).

Determination of relevant soil and environmental parameters at a given site can be labor-intensive and time-consuming but is inevitable when assessing the favorability of an environment for the presence of *Metarhizium* and *Beauveria* spp. Recent research provides strong evidence that plant communities, through their ecological indicator values (EIVs), are reliable and informative proxies for a wide range of environmental conditions, particularly soil properties and moisture gradients (20). EIVs are widely used in vegetation assessments to characterize local abiotic conditions, such as soil moisture, pH, nutrient availability, and temperature (21–23). This system represents an expert-derived, categorical assessment of the environmental preferences of individual plant species when growing in competition with other plant species in multispecies plant communities. It is averaged across species within plots to serve as bioindicators of local site conditions (24). Landolt indicator values (25), which represent one type of EIV, have been demonstrated to be particularly effective and informative to infer environmental conditions in Switzerland and the European Alps (26). Landolt indicator values might provide an efficient and easily applicable tool to assess and characterize soil and environmental parameters, and have potential as a tool to indirectly determine or even predict favorability of a given site for the presence of fungal taxa such as *Metarhizium* and *Beauveria* spp. However, associations among the presence of *Metarhizium* and *Beauveria* spp., soil and environmental parameters, and indicator values have remained unaddressed.

While community-level approaches such as EIVs offer insights into broader site conditions, species-level analyses, such as indicator species analysis (ISA), may help identify specific associations among individual plants and particular environmental conditions or microbial species. ISA has recently been proposed to identify potential pathogens in complex plant–disease systems (27). This is particularly interesting in the light of the endophytic and root-associated lifestyle of some entomopathogenic fungal species such as *M. brunneum*, *M. robertsii*, or *B. bassiana* (4). Interactions of these fungi with plants can result in a variety of beneficial effects and system services for the host plant, including resilience to plant-feeding insects, increased plant growth, or stress tolerance (3, 5). This close association with plants further highlights the potential of using plants and plant-based indicators to understand the ecological niches or even specific interactions between single plant species and entomopathogenic fungi.

The presence and persistence of natural populations of *Metarhizium* spp. and *Beauveria* spp., as well as the successful establishment of fungal BCAs based on isolates of these genera, depend on a complex of many factors, including soil physicochemical properties, climate factors, and plant interactions, in addition to the availability of suitable hosts. Permanent grasslands represent interesting habitats for investigating these interactions, as they typically harbor diverse populations of these fungi and provide an environment without soil management such as tilling and crop rotation (18). In this study, we surveyed 72 real-world grassland sites representing three different management types in lowland as well as subalpine regions in Switzerland and explored the occurrence of *Metarhizium* spp. and *Beauveria* spp. in relation to environmental factors as well as plant communities. Our specific goals were to (i) investigate and describe the diversity and prevalence of *Metarhizium* spp. and *Beauveria* spp. in Swiss grassland soils representing areas of two different climatic/geographic regions (i.e., lowland or subalpine) under different management regimes (i.e., extensive, low-intensive, or intensive); (ii) assess the link between occurrence of these fungal taxa with soil parameters (e.g., pH, texture, organic matter, and nutrients) and climatic conditions; (iii) explore correlations with plant community composition and plant species based on Landolt indicator values; and (iv) apply indicator species analysis to identify potential plant species linked to fungal presence. By investigating correlations among all the different factors, we provide an initial insight into a possible use of EIVs and indicator plant species to characterize the favorability of soil environmental conditions for the presence or establishment of *Metarhizium* and *Beauveria* populations.

## MATERIALS AND METHODS

### Study design

The study was performed on soil samples collected and processed by Fox et al. (28) and included samples from 72 grassland sites in Switzerland. One half of the sites are located in the Swiss lowlands and the other half in subalpine regions. Sites comprised three management types: intensively managed, low-intensively managed, and extensively managed grassland. Soil samples (ø 2.5 cm, depth 20 cm) were collected from each corner of four crosswise arranged 2 m × 2 m subplots. Two subplots were placed 5 m from the field center along one direction, and the other two subplots were placed 10 m from the center along the perpendicular direction (28, 29). The resulting 16 soil cores were combined, homogenized, sieved (2 mm), and freeze-dried (28). Within the 4 m^2^ subplots, plant species present were identified, and the percentage of coverage of every species was estimated. Soil sampling and vegetation assessment were performed in the summer months of 2017 at the peak of the vegetation season (28).

### Environmental, management, and Landolt indicator values

Climatic factors, including mean temperature (mean annual temperature), mean precipitation (mean annual precipitation), mean solar radiation (mean annual solar radiation), and mean sunshine hours (mean annual sunshine hours), were received from Meteo Swiss. Abiotic soil factors, including soil pH, total soil phosphorus, carbon, and nitrogen, as well as soil texture (% sand, silt, and clay), were obtained from Fox et al. (28). In addition, total soil organic matter content, available soil phosphorus (mg/100 g), and bulk soil density (g/cm^3^) were determined using loss on ignition, the Swedish method described by Egnér et al. (30), and the ISO 11272:2017 standard method, respectively. Climate and abiotic soil factors are collectively referred to as environmental factors in this study (Table S1).

Sites were chosen with the aim of covering a large gradient of management intensities. Extensive and low-intensive sites refer to plots belonging to eco-schemes according to the Swiss Direct Payment Regulation SR910.13. Extensive management defines sites with a total ban of fertilization, combined with a late first utilization and a plant species composition indicating special ecological quality (nutrient-poor, species-rich grasslands). Low-intensity management refers to plots with no or a very low fertilizer input combined with a late first utilization but with no specific requirements for plant species composition. Intensive management denominates sites which do not belong to an eco-scheme.

The Landolt indicator values for reactivity (R), nutrients (N), temperature (T), and moisture (F) were assigned to each identified plant species, following the categorization in *Flora Indicativa* (25). Subsequently, values for the four Landolt indicator values were calculated as weighted averages, i.e., of T, R, or N, and F indicator values relative to coverage of all the plant species present per site. Landolt-R reflects soil reactivity, essentially indicating soil pH; Landolt-N describes the availability of key nutrients, namely nitrogen, phosphorus, and potassium; Landolt-T captures climatic temperature gradients, such as the differences between lowland and subalpine habitats; Landolt-F is a proxy for soil moisture conditions. All indicator values range from 1 to 5, where 1 corresponds to acidic, nutrient-poor, cold, or dry conditions, and 5 corresponds to alkaline, nutrient-rich, warm, or moist conditions.

### Soil samples and DNA extraction and metabarcoding

Soil DNA extraction, PCR amplification, and subsequent sequencing were performed by Fox et al. (28). Briefly, DNA was extracted from 0.3 g of soil from each sample as described by Hartmann et al. (31), and fungal ITS2 regions were amplified in three replicates using CS1/CS2-tagged ITS3 and ITS4 primers (32). Pooled PCR replicates were sequenced on an Illumina MiSeq v3 platform at the Génome Québec Innovation Center (Montréal, Canada). Raw sequences were deposited by Fox et al. (28) in the NCBI SRA archive under accession number PRJNA641340.

### Sequence filtering

Raw data of ITS sequence reads were reanalyzed in the frame of this study using a pipeline adapted from Manoni et al. (33) and Herzog et al. (34). We applied a pipeline for quality control that was based on VSEARCH, including PCR primer trimming with *Cutadapt* (allowing for one mismatch), PhiX contamination removal using *bowtie2* to align sequences to a PhiX reference, and quality filtering with the *fastq_filter* function, allowing for a maximum expected error of one. Following the quality control filtering, we employed the DADA2 R script for amplicon sequence variant (ASV) inference (35). This process started with error rate learning from the filtered sequences (*learnErrors*), followed by dereplication (*derepFastq*) and variant inference (*dada*) for both forward and reverse reads. Paired-end reads were then merged (*mergePairs*) in which mismatches were not allowed. The final step in DADA2 generated a sequence table from the merged sequences. To refine the data, fungal sequences shorter than 150 bp were removed (*seqkit*). Next, chimeric sequences were identified and removed (*uchime3_denovo*). Remaining ASVs were tested for ribosomal signatures (*ITSx*), and unsupported sequences were discarded. Taxonomic assignment was performed using MOTHUR, where sequences were matched against the UNITE v8.3 database (36), with a classification cutoff of 80% identity.

### Phylogeny

Taxonomic classification of fungal ASVs based on the UNITE database was supplemented with a sequence similarity search using VSEARCH. Each ASV was compared to a custom reference database (95% identity threshold) containing 10 representative sequences of the genera *Metarhizium* and *Beauveria*. ASVs identified by either method were subsequently aligned using BioEdit (v7.2.5) alongside sequences of 20 reference strains of *Metarhizium* and *Beauveria* spp. ASVs falling outside the genus boundaries based on alignment were excluded. The resulting alignments were used to construct a maximum-likelihood phylogenetic tree in MEGA X (v11.0.9), applying the Tamura-Nei substitution model with 1,000 bootstrap pseudoreplicates.

### *Metarhizium* clade 1 real-time PCR quantification

DNA was purified using Zymo-spin III HCR columns, following the manufacturer’s protocol. DNA concentration was measured with a Cary Eclipse fluorescence spectrophotometer (Varian Inc., Palo Alto, CA) using PicoGreen fluorescent nucleic acid stain (Invitrogen, Carlsbad, CA), and subsequently adjusted to 2.5 ng/µL. Quantification of *Metarhizium* clade 1 was performed according to the protocol described by Schneider et al. (37). qPCR data were analyzed in R (R Core Team, 2023) using descriptive statistics, and a Spearman correlation test was performed between the qPCR data and cumulated sequence counts for ASVs included in *Metarhizium* clade 1 and displayed as a scatterplot with linear regression.

### Environmental factor, management, and Landolt indicator value statistics

Statistical analyses were performed in R (38). The sequence counts for all fungal ASVs were transformed into relative abundance per site to stabilize the variance across sites. Wilcoxon rank-sum tests were used to compare the relative abundance per site of *Metarhizium*- and *Beauveria*-ASVs between the lowland and subalpine regions, and Kruskal–Wallis tests were used to assess the relationship between ASV abundance and management type (extensive, low-intensive, intensive). Boxplots were generated for both analyses using *ggplot2*.

Environmental factors (Table S1), including four climate factors (mean temperature, mean precipitation, sunshine hours, and solar radiation) and nine abiotic soil factors (pH, total soil organic matter, available phosphorus, total phosphorus, carbon-to-nitrogen ratio, percentage of sand, silt, and clay, and bulk soil density), were analyzed using diagnostic plots to assess normal distribution, homoscedasticity, and outliers. Spearman’s rank correlations were calculated between all pairs of variables to examine the relationship among these factors. The same analysis was applied to the management factor (total applied nitrogen) as well as to the four Landolt indicator values (R, N, T, and F).

The relationship between relative ASV abundance and all environmental and management factors, as well as Landolt indicator values, was assessed using Spearman’s rank correlation; *P*-values were adjusted for multiple testing using Benjamini–Hochberg correction. The correlations were visualized in a heatmap colored by strength and direction of the associations.

### Generalized linear models

A generalized linear model (GLM) was fitted using the binomial family to investigate the relationship between the binary presence/absence of the *Metarhizium* and *Beauveria* ASV and the set of environmental and management factors, as well as Landolt indicator values, which were incorporated as predictors. The interfactor correlations for all variables were below 0.8, indicating no strong multicollinearity. An exception was the correlation between Landolt-N and Landolt-F, which exceeded this threshold; consequently, the F indicator was excluded from subsequent analyses. All factors enclosed in the analysis were scaled using the *scale* function, and no factors were excluded due to multicollinearity, as all variance inflation factors (VIFs) were below 10. A stepwise model selection procedure was applied to refine the model, beginning with backward selection from a full model, followed by forward selection starting with a null model, and a final bidirectional selection using the *step* function. The best model, based on Akaike Information Criterion (AIC), was selected, and model diagnostics were performed using standard residual diagnostic plots (normal distribution, homoscedasticity, and outliers). To assess predictive performance, predicted probabilities of presence were computed using the *predict* function. These predictions were compared to actual outcomes via a receiver operating characteristic (ROC) curve, and the area under the curve (AUC) was calculated to evaluate model discrimination. To estimate generalizability, a 10-fold cross-validation was implemented using the *boot* package and the *cv.glm* function.

### Plant community structure and indicator plant analysis

Bray–Curtis dissimilarity on the plant community data was calculated using the *vegdist* function from the *vegan* package. The Bray–Curtis dissimilarity matrix of the plant communities was then correlated with environmental and management factors, as well as with the *Metarhizium* and *Beauveria* ASVs, using the *adonis* function with 999 permutations. Principal Coordinates Analysis (PCoA) was performed on the Bray–Curtis dissimilarity matrix to reduce the dimensionality of the community data. The first two principal coordinates (PC1 and PC2) were extracted, and plots were generated to visualize the plant community structure, colored according to lowland and subalpine locations or management intensity. Spearman’s rank correlations were calculated between each *Metarhizium* and *Beauveria* ASV and the first two PCs of the plant community. ASVs significantly correlating with PC1 or PC2 (|Rho| > 0.3) were highlighted in the PCoA plots, with arrows indicating the direction and strength of the correlation.

Indicator species analyses were conducted to identify plant species specifically associated with the presence of *Metarhizium* and *Beauveria* ASVs. Analyses were focused on plant species present at more than 10 sites. First, the *indval* function from the *labdsv* package was used to compare the presence/absence of ASVs with the presence/absence of plant species. Next, the *multipatt* function with *IndVal.g* was applied to compare ASV presence/absence with values of plant coverage (percent area covered by a species per site). Finally, Spearman’s rank correlation tests were performed between relative ASV abundance and plant coverage. *P*-values were adjusted for multiple comparisons using the Benjamini–Hochberg (BH) correction. Plant species were considered indicators when significant and above the threshold (indicator values > 0.5; correlations > 0.3) in all three analyses.

## RESULTS

### Four *Metarhizium* and two *Beauveria* ASVs are frequent in Swiss grassland soils

**Fig 1:**
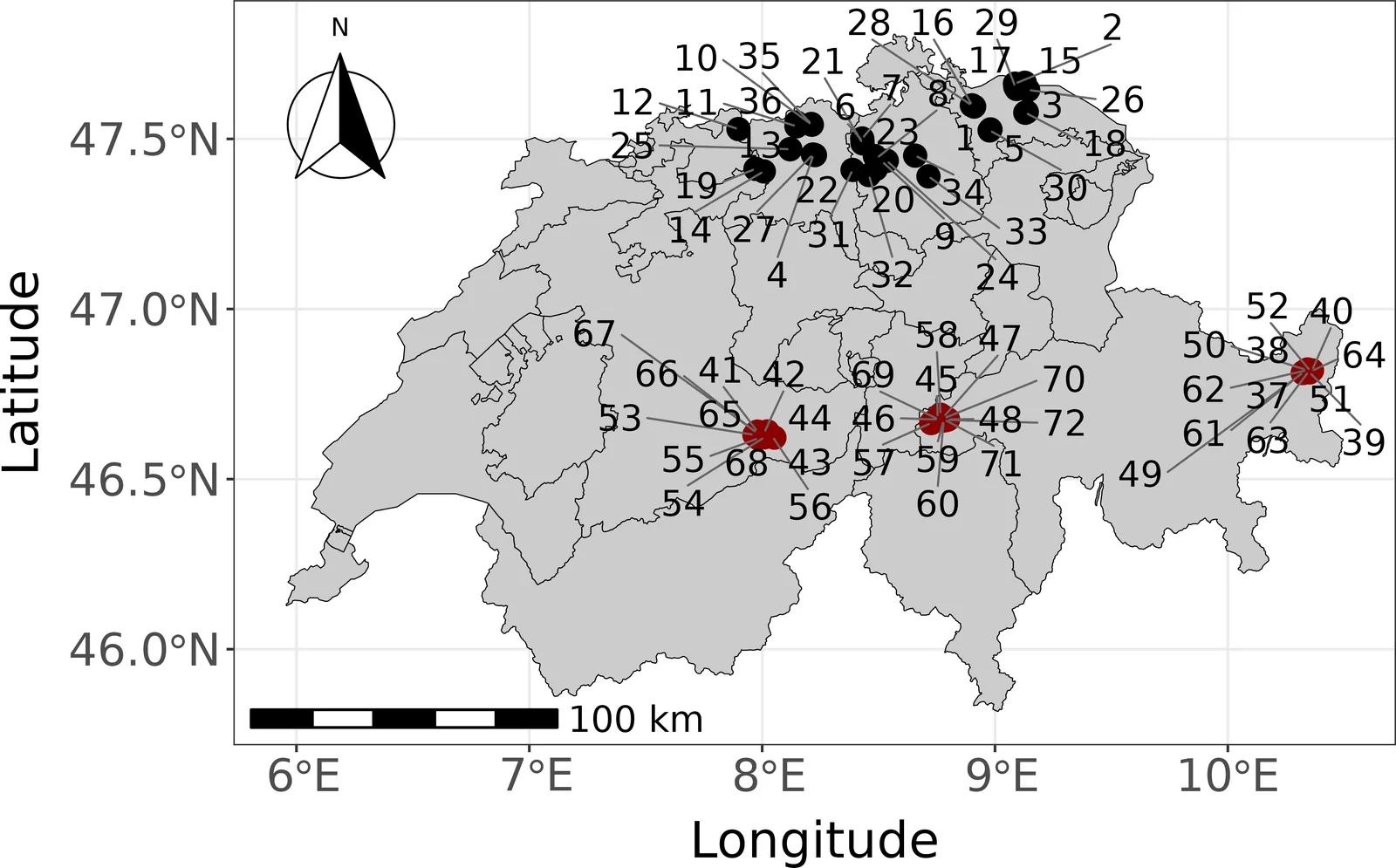
Map of Switzerland (from GADM) showing cantonal borders and the locations of the 72 study sites (28). Lowland sites are indicated by black circles, and subalpine sites by red circles.

**Fig 2:**
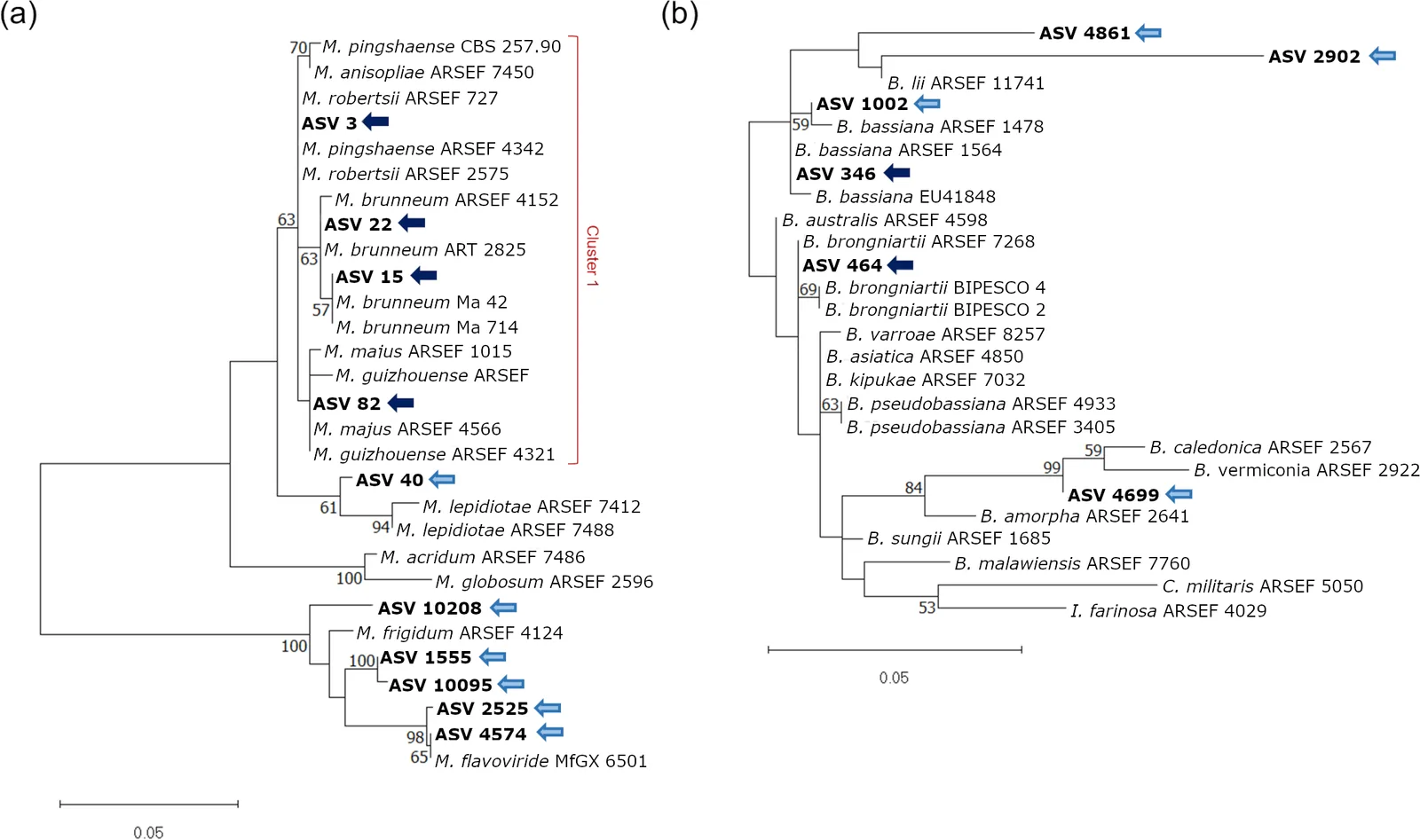
Maximum-likelihood phylogenetic tree based on alignments of ASVs representing identified (**a**) *Metarhizium* spp. and (**b**) *Beauveria* spp. ASVs, as well as 19 and 20 reference sequences, respectively. Bootstrap values are calculated with 1,000 pseudoreplicates, and values above 50% are shown at the branches. Frequent ASVs (9 to 44 sites) are labeled with dark blue arrows, rare ASVs (1 to 5 sites) with light blue arrows, and cluster 1 is indicated in red.

Amplicon sequencing data from 72 grassland sites in Switzerland (Fig. 1) were assessed for the presence and diversity of entomopathogenic fungi of the genera *Metarhizium* and *Beauveria*. Analyses revealed 11,976–29,896 fungal sequences per site, which were clustered into 9,269 fungal amplicon sequence variants (ASVs), with sampling depth close to saturation (Fig. S1). Ten and six of the fungal ASVs were assigned to the genera *Metarhizium* and *Beauveria*, respectively (Fig. 2). Six *Metarhizium* and four *Beauveria* ASVs were present at one to five sites only and, due to this low frequency, were excluded from further analyses (Fig. S3). The remaining four *Metarhizium* ASVs were present at 29–44 sites and were identified as *M. robertsii*/*M. pingshaense* (ASV 3), *M. brunneum* (ASV 15 and ASV 22), or *M. majus*/*M. guizhouense* (ASV 82). The two maintained *Beauveria* ASVs were detected at 9 and 10 sites and assigned to *B. bassiana* (ASV 346) ASV and *B. brongniartii* (ASV 464) (Fig. 2). The four frequent *Metarhizium* ASVs clustered within *Metarhizium* clade 1, which includes all currently commercialized BCA strains of this genus in Europe. Quantification of clade 1 using real-time PCR (qPCR) revealed 10^4^ to 10^8^ ITS copies per gram of soil dry weight, with two of the 72 sites below the detection threshold of 10^4^ copies per gram of soil dry weight (Fig. S2a). Cumulative ITS sequence abundance of the four *Metarhizium* ASVs correlated significantly (*R*^2^: 0.79, *P*: 3.3 *e*^−16^) with the qPCR-based data (Fig. S2b).

### Even versus regionally restricted occurrence of *Metarhizium* and *Beauveria*

**Fig 3:**
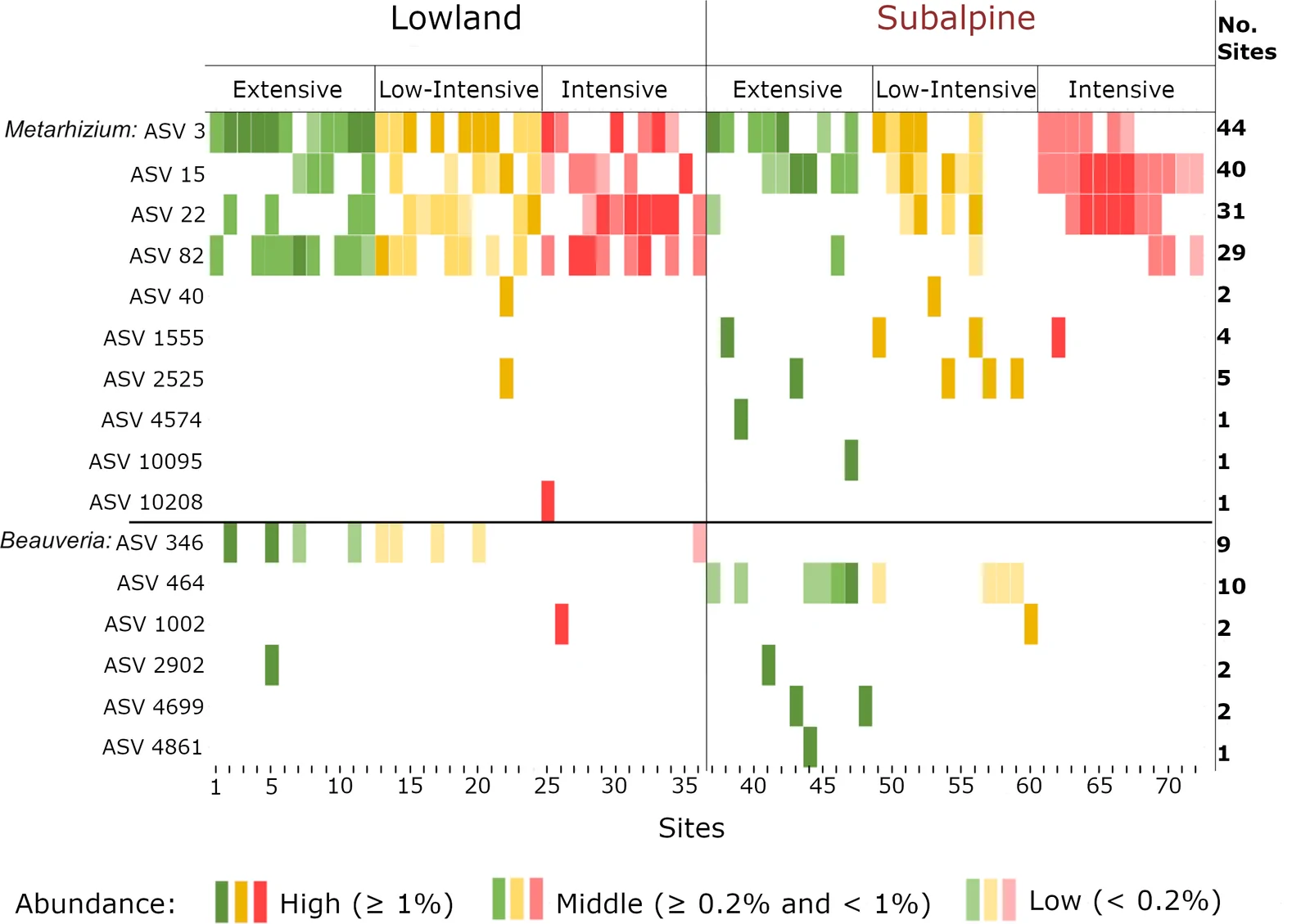
Frequency and relative abundance of *Metarhizium* and *Beauveria* ASVs at the 72 grassland sites. Sites with no presence in white, low abundance (<0.2% of all sequences per site) is shown in light color, medium abundance (≥0.2% and <1% of all sequences per site) is shown in medium color, and high abundance (≥1% of all sequences per site) is shown in dark color. The number of sites (No. sites) in which the respective ASVs are present are indicated in bold. Management intensity is indicated in different colors: extensive (green), low-intensive (yellow), and intensive (red). Sites 1–36 are in lowland, and sites 37–72 are in subalpine regions.

**Fig 4:**
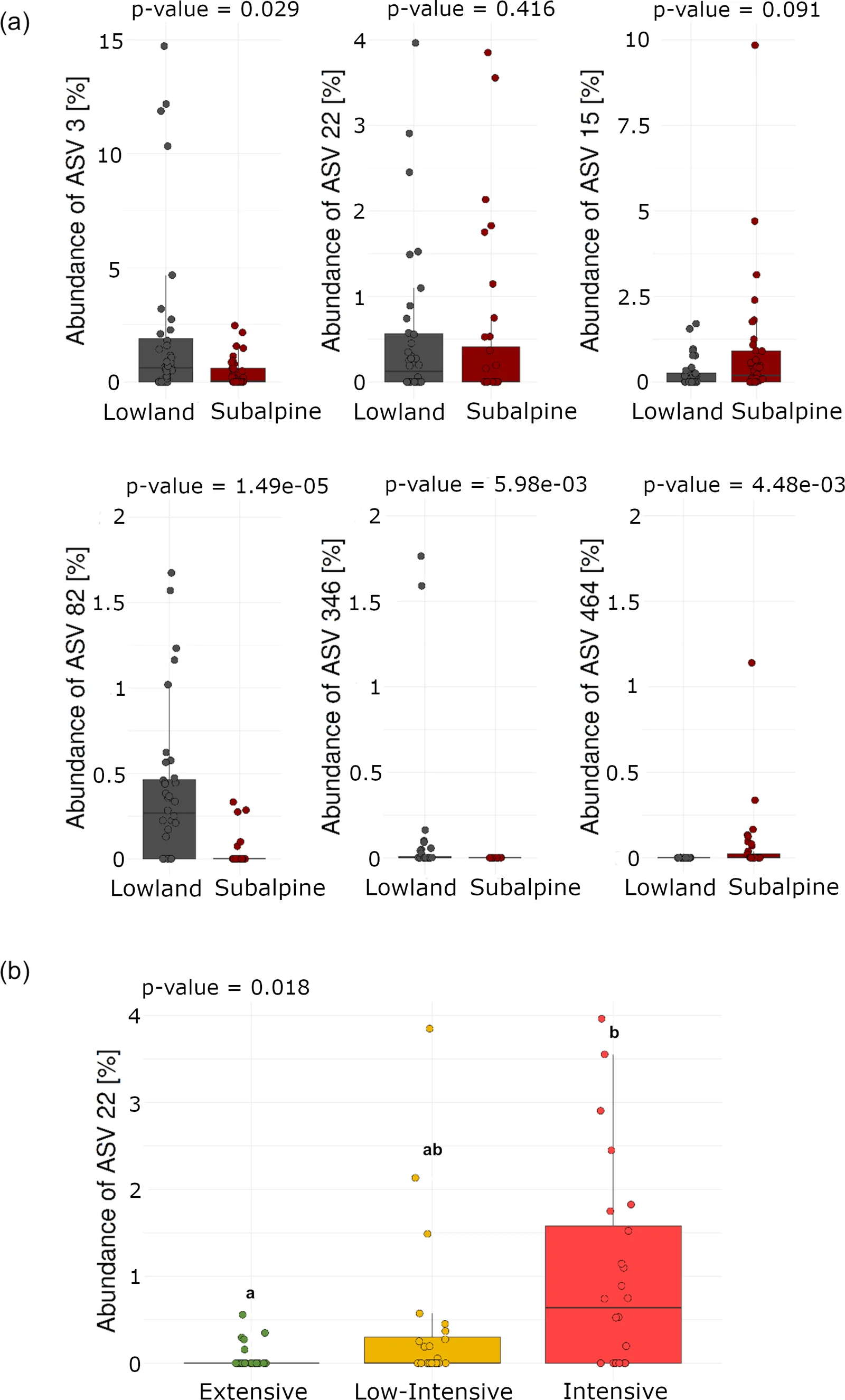
Boxplots of relative sequence abundance of the six frequently occurring ASVs, with *P*-values adjusted for multiple testing. (**a**) Comparison between lowland and subalpine grassland and (**b**) relative abundance of ASV 22 in extensively, low-intensively, and intensively managed grasslands. Significance is indicated by different letters.

We observed distinct presence/absence and relative abundance patterns among the four *Metarhizium* and two *Beauveria* ASVs across the 72 grassland sites (Fig. 3 and 4a). *M. robertsii*/*M. pingshaense* ASV 3 was the most frequent (present at 44 sites) and the most abundant, occurring at 19 sites with a relative sequence abundance above 1% and at four of these sites with a relative abundance above 10%. This was followed by *M. brunneum* ASV 22 and ASV 15 for which relative sequence abundances exceeded 1% at 12 and 10 sites, respectively. Although *M. robertsii*/*M. pingshaense* ASV 3 was present at 18 subalpine sites, its relative abundance was significantly lower in this region, ranging from 0.06% to 2.5% compared to 0.07% to 14.7% in the lowland sites (Fig. 4a). In contrast, *M. brunneum* ASVs 15 and 22 exhibited no differences in relative abundance between lowland and subalpine sites. On average, the relative sequence abundances for *M. robertsii*/*M. pingshaense* ASV 3 and *M. brunneum* ASVs 15 and 22 were 2.1%, 1.0%, and 1.1%, respectively, at the sites where they were present. These three ASVs were also the most frequent and evenly distributed, occurring at 43–61% of all sites and present in at least one-third of both lowland and subalpine grassland sites (Fig. 3).

*B. brongniartii* ASV 464 was found exclusively in subalpine grassland, whereas *B. bassiana* ASV 346 was detected only in lowland grassland and *M. majus*/*M. guizhouense* ASV 82 was found predominantly in lowland grassland (Fig. 3 and 4a). The average relative sequence abundances for ASVs 82 (*M. majus*/*M. guizhouense*), 346 (*B. bassiana*), and 464 (*B. brongniartii*) were 0.5%, 0.4%, and 0.2%, respectively, and at none of the sites was the relative sequence abundance above 1.8%. No significant differences in relative abundance of the ASVs relative to management intensity were found, except for *M. brunneum* ASV 22, which showed a significantly higher relative sequence abundance in intensively managed grasslands, paired with a lower presence in low-intensively and extensively managed sites (Fig. 3 and 4b).

### *Metarhizium* and *Beauveria* ASVs correlate with different environmental and management factors

**Fig 5:**
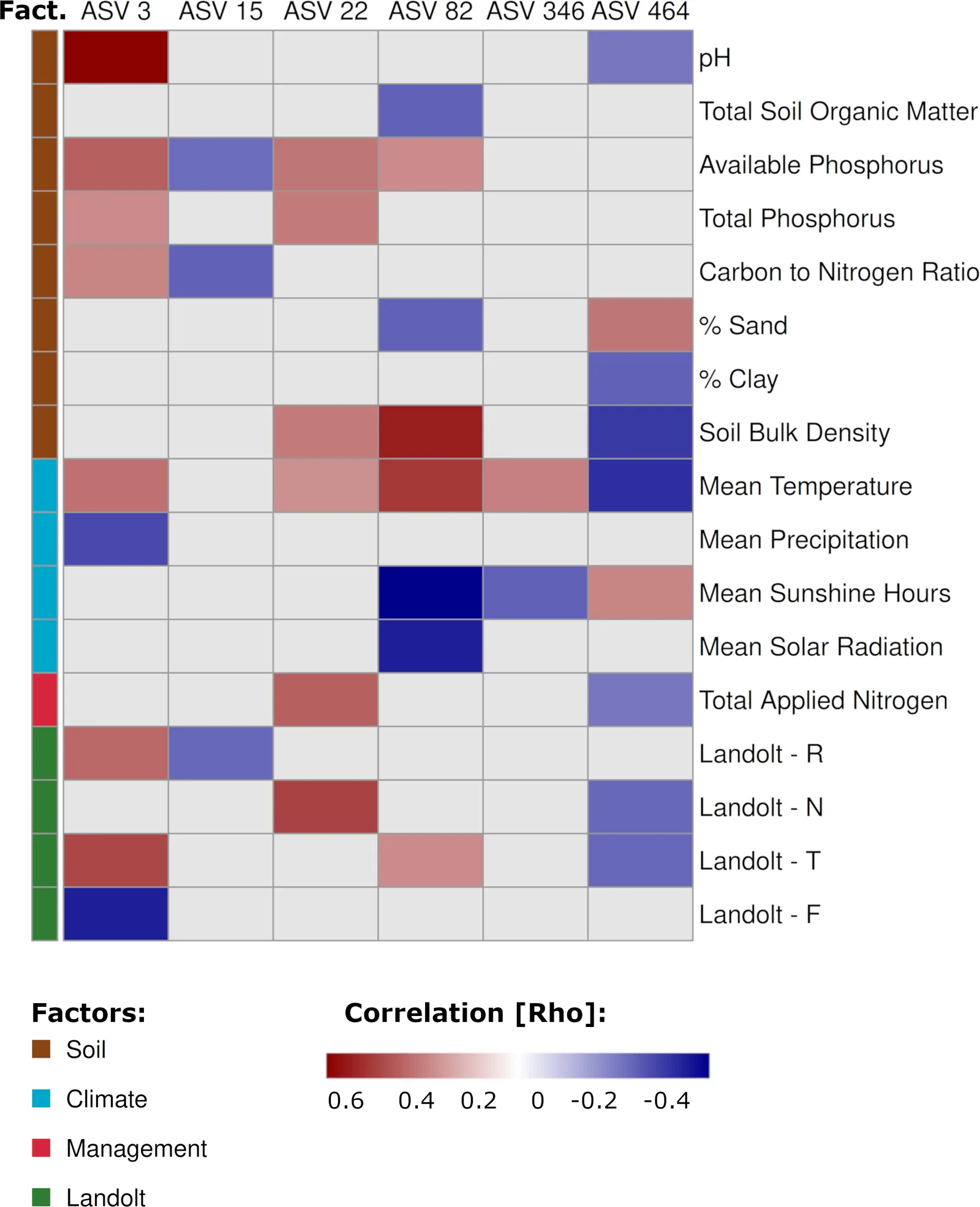
Heatmap of Spearman correlations between *Metarhizium* and *Beauveria* relative ASV abundances and abiotic soil, climate, and management factors (Fact.) as well as Landolt indicator values. Positive correlations are shown in red, negative correlations in blue, and not-significant correlations in white. Color gradients indicate strength of correlation.

Fourteen of the sixteen soil, environmental, or management factors showed a significant correlation with the relative sequence abundance per site of at least one of the prevalent *Metarhizium* or *Beauveria* ASVs (Fig. 5). The highest number of correlations was found for mean temperature, which correlated with five of the six ASVs (ASVs 3, 22, 82, 346, and 464), followed by available phosphorus, which correlated with four ASVs (ASVs 3, 15, 22, and 82). Individual ASVs revealed distinct correlation patterns showing positive or negative correlations to two (ASVs 15 and 346) or up to 10 factors (ASV 464). The strongest positive correlation was found between *M. robertsii*/*M. pingshaense* ASV 3 and pH (Rho: 0.64, *P*: 4.0*e*^−07^). In addition, *M. robertsii*/*M. pingshaense* ASV 3 showed weaker yet still significant correlations with soil nutrients (available P, total P, and C:N ratio, with Rho between 0.30 and 0.41) and temperature (Rho: 0.36, *P*: 1.4 *e*^−02^) and a negative correlation with mean precipitation (Rho: −0.40, *P*: 4.5 *e*^−03^) (Fig. 5). Correlation patterns were different even between ASVs 15 and 22, which belong to the same species, *M. brunneum*. While *M. brunneum* ASV 22 correlated with the soil nutrients (available P, total P, total applied N, with Rho between 0.33 and 0.39), as well as temperature (Rho: 0.29, *P*: 4.2*e*^−02^) and bulk soil density (Rho: 0.35, *P*: 1.7*e*^−02^), *M. brunneum* ASV 15 correlated negatively with only two environmental factors, i.e., available P and C/N ratio (Rho: −0.32 and −0.34, respectively).

The three ASVs 82 (*M. majus*/*M. guizhouense*), 346 (*B. bassiana*), and 464 (*B. brongniartii*), which exhibited characteristic geographic distributions between lowland and subalpine regions, all correlated with mean temperature (Rho: 0.50, 0.32, and −0.46, respectively) and mean sunshine hours (Rho: −0.58, −0.34, and 0.31, respectively), two factors that are distinctive for lowland and subalpine regions with significantly different altitudes. While ASVs 82 (*M. majus*/*M. guizhouense*) and 346 (*B. bassiana*), which were present and abundant particularly in the lowlands, correlated positively with mean temperature and negatively with mean sunshine hours, *B. brongniartii* ASV 464, present only in subalpine regions, correlated negatively and positively with the two factors, respectively. Furthermore, *M. majus*/*M. guizhouense* ASV 82 correlated negatively with solar radiation (Rho: −0.50, *P*: 2.4e^−04^) and positively with bulk soil density (Rho: 0.56, *P*: 2.4e^−05^), whereas *B. brongniartii* ASV 464 correlated negatively with bulk soil density (Rho: −0.44, *P*: 1.4e^−03^). Both factors, solar radiation and bulk soil density, are also characteristic for the differences between lowland and subalpine regions, respectively. In addition, the *B. brongniartii* ASV 464 correlated negatively with total applied nitrogen (Rho: −0.29, *P*: 4.5e^−02^).

### Generalized linear models reveal key predictors of fungal ASV presence

In addition to the correlation analyses of relative ASV abundance per site with individual environmental factors, a binary generalized linear model (GLM) was used to investigate environmental factors as predictors of the presence/absence of the four *Metarhizium* and two *Beauveria* ASVs. The forward and backward predictor selection process further reinforced the connection between *M. robertsii*/*M. pingshaense* ASV 3 presence and pH, as the final model identified only pH as a significant predictor of ASV 3 presence (Table 1). In contrast, models for the two *M. brunneum* ASVs (ASVs 15 and 22) revealed only a moderate model fit (AIC in Table S2). The model for *M. brunneum* ASV 15 included pH, available phosphorus (P), and temperature as predictors, with pH emerging as the only significant factor. In contrast, the model for *M. brunneum* ASV 22 identified total phosphorus and bulk soil density as significant predictors. The models for the three fungal ASVs that were differentially distributed between lowland and subalpine grasslands selected four factors associated with this distribution. The *M. majus*/*M. guizhouense* ASV 82 model included bulk soil density and mean solar radiation, and both factors resulted as significant predictors, together explaining 41% of the variation in *M. majus*/*M. guizhouense* ASV 82 presence. The *B. brongniartii* ASV 464 model incorporated temperature and bulk soil density as predictors and identified both factors as significant predictors explaining 50% of the variation for ASV 464 presence. While the models for *M. majus*/*M. guizhouense* ASV 82 and *B. brongniartii* ASV 464 showed a strong model fit, the model for *B. bassiana* ASV 346 was significant, although the selected predictor, mean sunshine hours, was not.

**TABLE 1:** Generalized linear models (GLMs) predicting the presence/absence (binary) of fungal ASVs^*a*^

	Predictor	*R* ^2^	*P*-value	*R*^2^ total	*P*-value total
ASV 3	pH	0.33	3.62e-06	0.33	1.71e-08
ASV 15	pH	0.04	0.048		
	Available phosphorus	0.05	0.061		
	Temperature	0.04	0.067	0.13	4.12e-03
ASV 22	Total phosphorus	0.06	0.023		
	Bulk soil density	0.08	9.80e-03	0.15	5.76e-04
ASV 82	Bulk soil density	0.14	2.12e-03		
	Solar radiation	0.19	6.86e-03	0.41	2.07e-09
ASV 346	Mean sunshine hours	0.25	0.111	0.25	2.25e-04
ASV 464	Temperature	0.22	0.021		
	Bulk soil density	0.11	0.026	0.50	5.02e-07

^*a*^ For each ASV, the table reports McFadden’s pseudo-*R*² and *P*-values for individual predictors, reflecting their contribution to the model, as well as total *R*² and overall model *P*-values from likelihood-ratio tests comparing the full model to a null model.

### *Metarhizium* and *Beauveria* ASVs correlate with plant communities and the derived ecological indicator values

**Fig 6:**
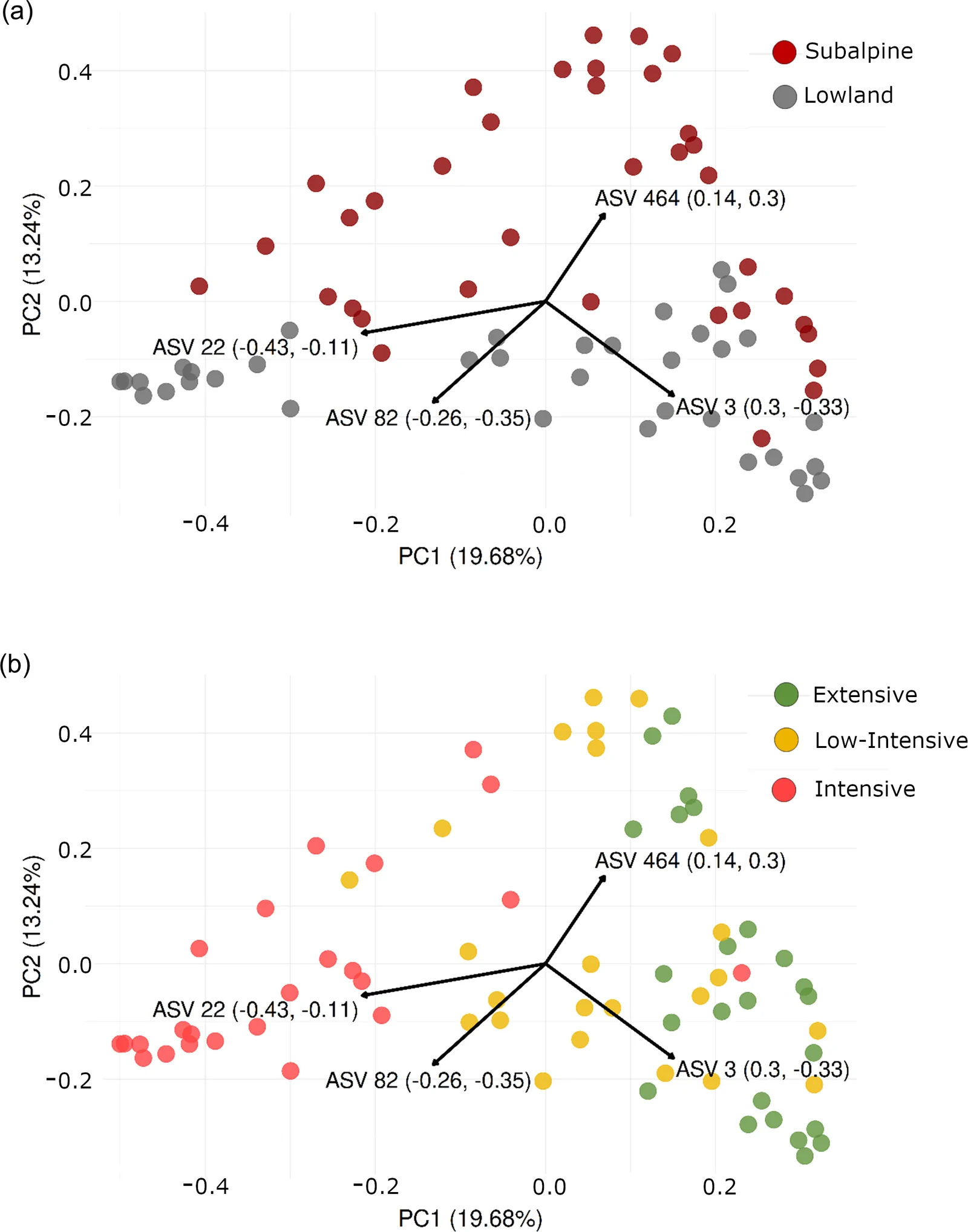
Principal coordinate plots of plant communities per site (**a**) colored according to subalpine (dark red) and lowland (grey) sites and (**b**) colored according to management intensity: extensive (green), low-intensive (yellow), and intensive (red). The percentage of variation explained by the plotted principal coordinates (PCs) is indicated on the axes. ASVs that correlate significantly with at least one of the PCs (Rho > 0.3) are shown with black arrows, with the Rho in brackets next to the ASV name.

Principal coordinate analysis (PCoA), with the first two axes explaining 19.7% (PC1) and 13.2% (PC2) of the variation in plant community structure, illustrates a clear separation of sites based on geographic region (Fig. 6a) and management intensity (Fig. 6b). The plant community composition significantly correlated with both geographic regions, as well as with management intensity, with the axes explaining 10% and 18% of the variance (Table S3). In a full model assessing how environmental and management factors relate to plant community structure, most variables showed a significant effect. These included soil pH, soil organic matter, all measured nutrients, sand and silt content, and bulk soil density (Table S3). In addition, two climate factors—temperature and solar radiation—as well as both management factors were significantly associated with plant community composition. All together, these factors explained 43% of the variance. Five of the six *Metarhizium* and *Beauveria* ASVs significantly correlated with plant community composition, with the exception of *B. bassiana* ASV 346 (Table S3). Specifically, *M. robertsii*/*M. pingshaense* ASV 3, *M. brunneum* ASV 22, *M. majus*/*M. guizhouense* ASV 82, and *B. brongniartii* ASV 464 showed significant correlations with at least one of the first two principal components (Fig. 6). While *M. majus*/*M. guizhouense* ASV 82 and *B. brongniartii* ASV 464 closely align with the plant community separation by geographic regions (Fig. 6a), *M. brunneum* ASV 22 and, to a lesser extent, *M. majus*/*M. guizhouense* ASV 82 and *M. robertsii*/ *M. pingshaense* ASV 3 correlated with the community separation by management intensity (Fig. 6b).

Four Landolt indicator values, i.e., R (reactivity), N (nutrients), T (temperature), and F (moisture), were calculated from the plant communities at each site. *M. robertsii/M. pingshaense* ASV 3 showed a positive correlation with the plant-derived Landolt-R (Rho: 0.39, *P*: 7.1e^−03^) and Landolt-T (Rho: 0.46, *P*: 7.4e^−04^) and a negative correlation (Rho: -0.36, *P*: 2.4e^−04^) with Landolt-F (Fig. 5). *M. brunneum* ASV 22 was positively correlated with Landolt-N (Rho: 0.47, *P*: 5.2e^−04^), while *M. majus/M. guizhouense* ASV 82 and *B. brongniartii* ASV 464 exhibited positive (Rho: 0.30, *P*: 3.8e^−02^) and negative (Rho: -0.33, *P*: 2.4e^−02^) correlations, respectively, with Landolt-T. *B. brongniartii* ASV 464 showing an additional negative correlation to Landolt-N (Rho: -0.32, *P*: 2.4e^−02^).

### Indicator plant species for *Metarhizium* and *Beauveria* ASVs

Indicator species analysis identified 18 plant species as potential indicators for four of the six frequent ASVs (ASVs 3, 82, 346, and 464), based on significant indicator values and correlation analyses (Table 2 and Table S4). Four plant species were associated with *M. robertsii*/*M. pingshaense* ASV 3, while only one indicator species was found for *M. majus*/*M. guizhouense* ASV 82. Furthermore, 10 indicator plant species were identified for *B. bassiana* ASV 346, with seven showing higher coverage per site in the lowland grasslands (Table 2). Similarly, five indicator plant species were identified for *B. brongniartii* ASV 464, with four showing higher coverage in the subalpine grasslands. While 16 of the identified plant species were indicative for one ASV, only two plant species, *Galium mollugo* and *Arrhenatherum elatius*, were indicative for two ASVs, i.e., ASVs 3 (*M. robertsii*/*M. pingshaense*) and 346 (*B. bassiana*).

**TABLE 2:** Indicator plant species for fungal ASVs and comparison of their coverage between lowland and subalpine sites^*a*^

Fungal ASV	Plant	Fungal presence/absence Plant presence/absence		Fungal presence/absence Plant coverage		Fungal abundance Plant coverage (Spearman)		Lowland (L)/subalpine (Sa) Plant coverage (Wilcox)	
		Indicator value	*P*-value	Indicator value	*P*-value	Rho	*P*-value	Higher abundance	*P*-value
ASV 3	*Galium mollugo*	0.66	0.001	0.85	0.001	0.66	3.04e-08	L > Sa	1.57e-03
	*Achillea millefolium*	0.55	0.002	0.84	0.001	0.45	5.79e-04	L = Sa	5.11e-02
	*Arrhenatherum elatius*	0.53	0.004	0.82	0.002	0.49	2.62e-04	L > Sa	6.68e-05
	*Helictotrichon pubescens*	0.50	0.001	0.73	0.001	0.51	1.40e-04	L > Sa	8.22e-03
ASV 82	*Poa trivialis*	0.51	0.003	0.76	0.002	0.47	7.02e-04	L > Sa	3.07e-05
ASV 346	*Medicago lupulina*	0.58	0.007	0.80	0.001	0.39	8.77e-03	L > Sa	1.45e-04
	*Knautia arvensis*	0.56	0.008	0.78	0.003	0.38	1.03e-02	L = Sa	8.98e-02
	*Ajuga reptans*	0.60	0.008	0.88	0.002	0.38	1.03e-02	L > Sa	1.10e-02
	*Prunella vulgaris*	0.61	0.010	0.84	0.003	0.38	1.03e-02	L > Sa	3.49e-03
	*Arrhenatherum elatius*	0.66	0.005	0.84	0.002	0.34	2.49e-02	L > Sa	6.68e-05
	*Bromus erectus*	0.67	0.002	0.82	0.002	0.40	7.99e-03	L = Sa	2.66e-01
	*Centaurea jacea*	0.60	0.003	0.81	0.001	0.48	8.01e-04	L > Sa	5.03e-05
	*Galium mollugo*	0.59	0.019	0.79	0.011	0.34	2.24e-02	L > Sa	1.57e-03
	*Sanguisorba minor*	0.60	0.005	0.78	0.003	0.41	7.99e-03	L = Sa	3.55e-01
	*Holcus lanatus*	0.74	0.001	0.75	0.013	0.36	1.36e-02	L > Sa	4.97e-07
ASV 464	*Silene vulgaris*	0.70	0.001	0.88	0.001	0.51	2.87e-04	L < Sa	2.42e-07
	*Festuca rubra*	0.60	0.044	0.86	0.001	0.44	1.55e-03	L < Sa	9.80e-04
	*Cynosurus cristatus*	0.55	0.004	0.74	0.006	0.44	1.55e-03	L = Sa	1.02e-01
	*Chaerophyllum hirsutum*	0.52	0.001	0.73	0.001	0.50	3.35e-04	L < Sa	1.85e-04
	*Agrostis capillaris*	0.53	0.004	0.76	0.001	0.43	1.85e-03	L < Sa	9.46e-07

^*a*^ Indicator species analysis and Spearman correlation were performed for the four *Metarhizium* and two *Beauveria* ASVs with plant species occurring at more than 10 sites. Indicator values were calculated using the presence/absence data of fungal ASVs and either the presence/absence of or percent area covered by a plant species per site. Spearman’s rho was calculated based on the relative sequence abundance of fungal ASVs and plant species coverage. *P*-values were adjusted for multiple comparisons using the Benjamini–Hochberg correction. Shown are plant species that were significant and above the threshold indicator values (>0.5) and the threshold correlations (>0.3) in all three analyses.

## DISCUSSION

The importance of entomopathogenic fungal species of the genera *Metarhizium* and *Beauveria* as regulators of arthropod populations is well recognized, and their presence in particular habitats is considered a beneficial factor for the natural self-regulation of pests. Subsequently, the ability to predict favorability of a particular environment or site for the presence of these fungi, or even for their establishment following application of selected strains in the context of biological control, is of great importance. In this study, as a first step toward this goal, we investigated associations of *Metarhizium* and *Beauveria* spp. with a range of factors, including soil physicochemical properties, climate factors, and plant interactions. We demonstrated the frequent presence and high relative abundance of both genera in an amplicon sequencing dataset including 72 Swiss grassland sites. We identified 10 *Metarhizium* and six *Beauveria* ASVs representing different species and strains. ASVs exhibited distinct presence/absence and relative abundance patterns that correlated with various climatic, management, and abiotic soil factors, indicating their adaptation to prevailing site conditions. The correlations were further reflected by plant-derived EIVs for reactivity, nutrient availability, temperature, and soil moisture, which were inferred from the plant communities. The demonstrated associations, as well as the single indicator plant species identified for specific fungal ASVs, highlight and reinforce the complexity of direct and/or indirect interactions of these fungi with their environment, but also offer potential for assessing the favorability for the presence and/or establishment of *Metarhizium* and *Beauveria*, or possibly other species.

### Diversity and taxonomic resolution of *Metarhizium* and *Beauveria* spp. in Swiss grasslands

The metabarcoding approach applied in this study targeted the ITS2 region of the ribosomal gene cluster, a locus generally used for cultivation-independent fungal diversity assessment. Previous studies have shown that the resolution of this marker within *Metarhizium* and *Beauveria* can be limited (39). Nevertheless, we reliably identified 10 distinct *Metarhizium* ASVs and six distinct *Beauveria* ASVs. Sequence variation among the *Metarhizium* clade 1 ASVs or the *Beauveria* ASVs was between one and four bases. It was possible to assign prominent ASVs to species such as *M. brunneum* (ASVs 15, 22), *B. bassiana* (ASV 346), and *B. brongniartii* (ASV 464). In the case of the prevalent ASVs 3 (*M. robertsii*/*M. pingshaense*) and 82 (*M. guizhouense*/*M. majus*), detected at 44 and 29 sites, respectively, allocation to single species was not possible. Cultivation-based approaches applying TEF barcoding, as well as microsatellite marker analyses, have shown that in Switzerland, as well as in northern Italy, Austria, and Denmark, *M. brunneum* and *M. robertsii* are the prevailing dominant *Metarhizium* species (18, 40–43). Therefore, it can be assumed that ASV 3 most likely represents *M. robertsii*, particularly since *M. pingshaense* has not been reported in Switzerland. In contrast, *M. guizhouense* and *M. majus* have both been reported in different European countries, i.e., *M. guizhouense* in Switzerland and Northern Italy, and *M. majus* in Denmark, which impedes allocation of ASV 82 to one of the two species based on the probability of reported occurrence. The eight rarely detected ASVs were assigned to species of the *M. flavoviride* complex, *M. lepidiotae*, *B. vermiconia*, *B. caledonica*, or *B. lii*, all of which, except for species of the *M. flavoviride* complex, have not been reported in Switzerland to date. Additional investigations, ideally using cultivation-based approaches, will be required to confirm the presence of these species at investigated sites.

### Distribution and soil physicochemical drivers of *Metarhizium* and *Beauveria* spp. populations

The four most frequent ASVs all belonged to *Metarhizium* clade 1, with *M. robertsii*/*M. pingshaense* and *M. brunneum* being particularly well represented, which confirmed previous cultivation-based reports (18, 40–43). The relative sequence abundance of the four *Metarhizium* ASVs was confirmed by the correlation of cumulative ITS sequences with qPCR-based data, which also supported the quantitative use of the sequence counts as a proxy for abundance. In general, *Metarhizium* spp. were more frequent and had a higher relative abundance in the grassland sites than *Beauveria* spp., reflecting findings from other studies conducted in different European countries (44–46). We found distinct presence/absence patterns among the different ASVs, with some detected at very few sites only, such as *M. lepidiotae-*related ASV 40, some present in both geographic regions (*M. robertsii*/*M. pingshaense* ASV 3 and *M. brunneum* ASVs 15 and 22), and some predominantly detected in the lowland (*M. majus*/*M. guizhouense* ASV 82, *B. bassiana* ASV 346) or subalpine (*B. brongniartii* ASV 464) grasslands. This strongly indicated a favorability of the different ASVs for certain ecological conditions or habitats.

With the comprehensive correlation and model-based analyses, we were able to identify environmental, management, and geographic factors characterizing the conditions relevant for the presence/absence or relative abundance of distinct ASVs. However, the effect of individual factors on different ASVs is complex, and differences were observed even between two *M. brunneum* ASVs (ASVs 15 and 22). While some factors correlated with the relative abundance per site of multiple ASVs, for instance, available phosphorus or mean temperature, other factors were associated with only one or two ASVs. For instance, based on correlation analyses, we identified pH as a characteristic factor for the favorability of *M. robertsii*/*M. pingshaense* ASV 3 and *B. brongniartii* ASV 464. This factor is well recognized to affect fungal community structures in grassland, arable land, and forests (47). Previous research with *M. robertsii* (formerly assigned to *M. anisopliae*) strain ARSEF 2575 (19) has demonstrated that lower pH can reduce expression of virulence factors and, therefore, potentially affect the infectivity of this fungus under ambient conditions in the field. In contrast, *B. brongniartii* ASV 464 was negatively correlated with soil pH, matching the lower mean pH in subalpine grasslands. Nevertheless, other mechanisms or factors might be more relevant for the occurrence of this species, such as temperature or bulk soil density. In parallel, we found that the Landolt-R indicator value, which reflects pH, shows a strong positive correlation with the relative abundance of *M. robertsii*/*M. pingshaense* ASV 3, indicating that this plant community-based value might qualify as a predictor of the relative abundance or presence of this ASV. However, it remains to be assessed to what degree this mechanism may directly or indirectly affect the abundance of taxa at the species or even within-species level in the field.

### Influence of land management and nutrient inputs

*M. brunneum* ASV 22 and *B. brongniartii* ASV 464 were both associated with the land-use intensity indicator “applied nitrogen.” While the first showed a positive correlation for nutrient-rich environments (applied nitrogen), the second, *B. brongniartii* ASV 464, was negatively correlated, matching its preference for extensively managed sites. Accordingly, the two ASVs correlated positively or negatively with Landolt-N. Interestingly, neither of the two ASVs revealed significant associations with C:N ratio. The C:N ratio has been reported as a driver for entomopathogenic fungi in various studies (16–18), and in our data, we detected a positive correlation with *M. robertsii*/*M. pingshaense* ASV 3 and a negative correlation with *M. brunneum* ASV 15. Other studies have shown that fungal ASV richness is higher in extensively managed Swiss pastures and that both management intensity and the associated Landolt-N indicator value influence fungal community structure (48–50). The two *M. brunneum* ASVs (15 and 22) showed contrasting associations to a number of factors, including the land-use intensity indicator applied nitrogen, the C:N ratio, as well as available phosphorus or mean temperature, as mentioned above. These differences indicate intraspecific adaptation and highlight the importance of investigating ecological associations not only at the species-level but also at the within-species level. Our findings revealed the importance of management practices and nutrient availability for shaping fungal populations. Similarly, other studies have reported effects of cover crops and tillage on *M. anisopliae* abundance in a feed grain crop rotation during the transition to organic agriculture (51) and a higher abundance of *Metarhizium* spp. in conventionally managed and fertilized vineyards than in organically managed vineyards that received organic fertilizers (17). However, in the latter study, fungi were assessed at the genus level, which may have masked species-level differences similar to those observed in our study.

### Geographic and climatic impacts on *Metarhizium* and *Beauveria* spp. occurrence

Fox et al. (28) have reported that regions with favorable growth conditions, i.e., lowland versus subalpine, have a significant but weaker effect on fungal communities than management intensity. Our findings suggest that some of the ASVs, namely *M. majus*/*M. guizhouense* ASV 82, *B. bassiana* ASV 346, and *B. brongniartii* ASV 464, mirror this community shift. The environmental factors associated with lowland versus subalpine differences are mainly climatic factors: temperature, which is generally lower, and mean sunshine hours and solar radiation, which are higher in subalpine areas, as well as bulk soil density, which is generally lower in subalpine soil. Accordingly, the three ASVs revealed positive or negative correlations with these factors. Two of these environmental factors were also selected in the GLM for *M. majus*/*M. guizhouense* ASV 82 (solar radiation, bulk soil density) and *B. brongniartii* ASV 464 (temperature, bulk soil density), supporting the associations. Since our analyses were performed on fungal ASVs isolated from soil, the factors “mean sunshine hours” and “mean solar radiation” may not have a direct effect on the presence/absence of the ASVs identified. However, temperature has been shown to be a crucial factor for the germination and growth of entomopathogenic fungi *in vitro* (12–14), suggesting that temperature is a key driver for the presence of these fungi in the warmer lowland or cooler subalpine regions, selecting for adapted taxa. The importance of temperature as a discriminating factor between lowland and subalpine biota was also reflected by differences in plant communities, translating into different Landolt-T values, which correlate positively or negatively with *M. majus*/*M. guizhouense* ASV 82 or *B. brongniartii* ASV 464, respectively.

### Host availability and its role in *Metarhizium* and *Beauveria* spp. distribution

We detected *B. brongniartii* ASV 464 in subalpine grasslands only. *B. brongniartii* is known to have a narrow host range, primarily infecting *M. melolontha* larvae, a pest in grassland soil in Europe. This fungus is available as a commercial biological control agent (BCA) against *M. melolontha*, and it has been demonstrated that the establishment and persistence of the fungal BCA depend not only on prevailing soil types but also on the presence of the host (44, 52, 53). Subalpine grasslands are the habitats and locations in which strong populations of *M. melolontha* are observed in Switzerland (54). For instance, large populations are known to exist in the southeast of Switzerland, a region included in this study, which corroborates the previously reported importance of host presence for the development of *B. brongniartii* (44). The presence of suitable hosts is important for the development of entomopathogenic fungal populations, not only as a source of nutrients but also for dispersal. Fungal mycelium can be transported and distributed by moving infected individuals, or spores on the body surface can be transported and horizontally transmitted by host arthropods (55–57). Most of the species detected in this study, such as *M. brunneum*, *M. robertsii*, or *B. bassiana*, have a broader host range. It is therefore difficult, if not impossible, to assess the complete host spectrum in soil at a given site and the effects that the presence/absence of individual hosts may have on the development of these fungi. However, as suggested by Bidochka et al. (58), habitat selection, rather than host insect selection, may primarily drive population dynamics in *Metarhizium*. This view is in line with our study, revealing the relevance of habitat characteristics such as geographic regions, management intensities, soil physicochemical and climatic factors, and associated plant communities. Future analyses applying cultivation-independent metabarcoding approaches (59) targeting soil arthropods in the context of the framework developed may allow us to investigate the importance of host diversity and abundance for *Metarhizium* and *Beauveria* population dynamics and further disentangle host versus habitat selection.

### Plant communities as proxies for environmental conditions

The effect of environmental and management factors on plant community composition is well recognized (22, 23), a view supported both by Mayerhofer et al. (49), who showed more homogeneous plant communities in intensively managed grasslands, and the results of our study. Moreover, we detected associations between five of the six prevalent *Metarhizium* and *Beauveria* ASVs and plant communities across the 72 sites. Subsequently, the Landolt indicator values R, N, T, and F, which are derived from the plant communities, showed strong correlations with several fungal ASVs. The same ASVs were also associated with corresponding measured environmental parameters, such as soil pH, nutrient levels, temperature, and precipitation. While the Landolt indicator values R and T are associated with the directly measurable factors pH and temperature, the Landolt indicator values N and F reflect complexes of several factors. Landolt-N originally referred to nitrogen only, but has been adapted and expanded to include and describe the status of a combination of nitrogen, phosphorus, and potassium (25). Landolt-F is even more complex as it is influenced not only by precipitation but also by site characteristics such as sunlight, topology, and soil composition. On one hand, complex Landolt indicator values such as N and F can offer a more comprehensive picture of ambient site conditions in one value, but on the other hand, they complicate the assessment of correlations with individual factors. One advantage of plant indicator values is that they integrate the site conditions over a time period of several years, as plant communities do not change abruptly. Our results suggest that plant-derived ecological parameters may serve as proxies for ambient environmental conditions and allow us to infer favorability of a given grassland site for particular *Metarhizium* or *Beauveria* taxa. Such a concept would save invasive soil sampling and time-consuming chemical soil analyses and allow on-site determination of environmental conditions. However, our results represent a first observation and thorough assessment of the detected associations, and performing, for instance, pot and greenhouse experiments under controlled conditions will be required to support and validate the results to pioneer such an approach.

### Indicator species and potential fungus–plant interactions

Fungus–plant associations were further revealed by the identification of 18 indicator plant species for individual *Metarhizium* or *Beauveria* ASVs. For instance, the two herbaceous plant species *Achillea millefolium* and *Galium mollugo* and the two grass species *Arrhenatherum elatius* and *Helictotrichon pubescens* were indicative of *M. robertsii*/*M. pingshaense* ASV 3, which aligns with findings reported by Wyrebek et al. (60), who commonly isolated *M. robertsii* from the roots of grasses and herbaceous plants. Interestingly, the majority of indicators identified were associated with the two *Beauveria* ASVs. Ten plant species were indicative of *B. bassiana* ASV 346, including two that also served as indicator species for ASV 3, i.e., *Arrhenatherum elatius* and *Galium mollugo,* and five for *B. brongniartii* ASV 464. Seven of the species associated with ASV 346 revealed negative correlations with altitude, while those linked to ASV 464 were mostly positively correlated, in accordance with the differential distribution of *B. bassiana* ASV 346 and *B. brongniartii* ASV 464 between lowland and subalpine grasslands (Table 2). However, it remains to be investigated how tightly linked the associations are, as according to the “Info Flora, 2025” (61), 13 of these indicator plant species occur across all the regions sampled in this study, both in lowland and subalpine grasslands.

The plant–ASV associations detected in this study may be based on co-occurrence, i.e., indicator plant species and fungal ASVs share similar environmental or management-related preferences, as discussed above, and/or they might reflect direct fungus–plant interactions. Such direct interactions between plants and entomopathogenic fungi such as *M. brunneum*, *M. robertsii*, or *B. bassiana* have been observed previously. Several studies have reported the preference of *Metarhizium* and/or *Beauveria* isolates for root-associated growth or endophytic colonization of certain groups of plants, e.g., monocots or certain wild plant species (60, 62–64). Furthermore, in a study of an agroecosystem with crop rotations and different cover crops, *M. robertsii* abundance was linked to plant identity (65). In contrast, in other studies, the existence of such associations has not been confirmed (41, 66). In this context, it also has been demonstrated that isolates of these entomopathogenic fungi, when growing in the rhizosphere or endophytically, interact with plants by supporting their resilience to plant-feeding insects, increasing plant growth and stress tolerance, or providing them with nutrients such as nitrogen (3, 67, 68). Further studies will have to verify the associations and interactions detected by investigating additional grasslands and other habitats, as well as experimentally testing the interactions under controlled conditions to gain a deeper understanding of the complex interactions among plants, fungi, and soil conditions. Nevertheless, our data support the potential of using plant species as ecological indicators of entomopathogenic fungal presence and distribution in soil ecosystems, independent of whether the link derives from sharing the same conditions and/or direct fungus–plant interactions.

### Conclusion

In conclusion, our results show that different species or taxa of *Metarhizium* and *Beauveria* are associated with grasslands that are characterized by distinct differences in geographic regions, management intensities, soil physicochemical and climatic factors, and plant communities. Associations of the taxa with different factors are highly complex, and to further investigate and unravel the complexity, it will be important to first cultivate isolates representing the different ASVs identified and to test effects of individual factors in controlled conditions. Correlations with plant community composition-derived EIVs (Landolt indicator values), as well as individual indicator plant species, further support the utility of vegetation data as a proxy for underlying environmental conditions influencing or indicating fungal presence and suggest potential functional interactions beyond shared habitat preferences. The results emphasize the importance of considering not only virulence but also environmental competence when developing, for instance, *Metarhizium* and *Beauveria* strains for biological pest control. The identification of environmental profiles conducive to the natural occurrence of *Metarhizium* and *Beauveria* spp. or the successful establishment of fungal BCAs is crucial in this context. Our findings indicate a potential to apply above-ground plant community assessment for identification of such profiles, circumventing invasive soil sampling and laborious soil analyses.

## Data Availability

Raw sequences were deposited by Fox et al. (28) in the NCBI SRA archive under the accession number PRJNA641340.

## SUPPLEMENTAL MATERIAL

Supplemental material aem.02513-25-s0001.docx Fig. S1 and S2; Tables S1 to S4.

ASM does not own the copyrights to Supplemental Material that may be linked to, or accessed through, an article. The authors have granted ASM a non-exclusive, world-wide license to publish the Supplemental Material files. Please contact the corresponding author directly for reuse.
