# Supplementary material for: Entomopathogenic Metarhizium and Beauveria species are associated with distinct geographic, management, and environmental factors, as well as plant communities
Source: Appl Environ Microbiol. 2026 Jul 21;92(8):e02513-25. doi: 10.1128/aem.02513-25 (PMC13488305; doi:10.1128/aem.02513-25)
Supplement: Supplementary file 1 [file aem.02513-25-s0001.docx]

**Supplements:**

Sequences

ASV richness

**Supplementary Figure 1:** Rarefaction curve for sampling depth of fungal communities with 11’976 – 29’896 fungal sequences per site.

(a)

(b)

**Supplementary Figure 2:** (a) *Metarhizium* clade 1 abundances based on ITS copy numbers determined with qPCR. b) Spearman correlation between ITS copy numbers determined with qPCR and summarized sequence counts of *Metarhizium* ASVs determined by sequencing.

**Supplementary Table 1:** Overview of all environmental, management and geographic factors as well as Landolt indicator values used in this study, with site-specific values provided for each variable.

|  |  | **Environmental Factors** | | | | | | | | | | | | | **Management Factors** | | **Geographic Factors** | | **Landolt Indicator**  **Values** | | | |
| --- | --- | --- | --- | --- | --- | --- | --- | --- | --- | --- | --- | --- | --- | --- | --- | --- | --- | --- | --- | --- | --- | --- |
|  |  | **Abiotic Soil Factors** | | | | | | | | | **Climate Factors** | | | |  | |  | |  | | | |
| **Site Number** | **Reference number of deposited sequences ^a^** | **pH** | **Total Soil Organic Matter** | **Available Phosphorus** | **Total Phosphorus** | **Carbon to Nitrogen Ratio** | **% Sand** | **% Silt** | **% Clay** | **Bulk Soil Density** | **Mean Temperature** | **Mean Precipitation** | **Mean Sunshine Hours Sun** | **Mean Solar Radiation** | **Management** | **Total Applied Nitrogen** | **Geographic Region** | **Altitude** | **Landolt - T** | **Landolt - R** | **Landolt - N** | **Landolt - F** |
| 1 | 2 | 7.9 | 8.36 | 8.66 | 119.81 | 16.66 | 17.5 | 52.2 | 30.3 | 0.65 | 9.83 | 1023.14 | 4.64 | 138.3 | Extensive | 0 | Lowland | 394.6 | 3.55 | 3.12 | 3.48 | 3.09 |
| 2 | 5 | 7.79 | 14.40 | 5.67 | 126.47 | 12.97 | 12.9 | 37.2 | 49.9 | 0.70 | 9.29 | 1113.26 | 4.73 | 138.89 | Extensive | 0 | Lowland | 442 | 3.45 | 3.15 | 3.42 | 2.91 |
| 3 | 6 | 7.73 | 11.81 | 2.14 | 88.80 | 13.63 | 15.2 | 35.9 | 48.9 | 0.78 | 9.33 | 1146.55 | 4.72 | 139.33 | Extensive | 0 | Lowland | 533.9 | 3.66 | 3.69 | 2.58 | 2.83 |
| 4 | 9 | 7.43 | 11.53 | 1.70 | 106.48 | 13.26 | 5.9 | 60.5 | 33.6 | 0.79 | 9.97 | 1074.65 | 4.87 | 138.45 | Extensive | 0 | Lowland | 431.5 | 3.53 | 3.58 | 2.41 | 3.11 |
| 5 | 18 | 7.53 | 7.60 | 3.96 | 58.82 | 19.63 | 35.4 | 39.3 | 25.3 | 0.86 | 9.65 | 995.09 | 4.81 | 138.7 | Extensive | 0 | Lowland | 416.8 | 3.55 | 3.67 | 2.45 | 2.92 |
| 6 | 24 | 8.01 | 6.99 | 11.23 | 104.07 | 16.79 | 17.1 | 47.7 | 35.2 | 1.01 | 9.43 | 983.87 | 4.81 | 136.29 | Extensive | 0 | Lowland | 455.5 | 3.27 | 3.14 | 3.38 | 2.36 |
| 7 | 29 | 6.86 | 8.09 | 1.63 | 40.04 | 11.39 | 28.5 | 32.8 | 38.7 | 0.87 | 8.41 | 1088.87 | 4.74 | 138.39 | Extensive | 0 | Lowland | 635.8 | 3.58 | 3.49 | 2.56 | 3.11 |
| 8 | 31 | 7.66 | 8.05 | 0.48 | 48.57 | 26.01 | 21.4 | 48.4 | 30.2 | 0.83 | 8.52 | 1104.84 | 4.72 | 138.12 | Extensive | 0 | Lowland | 583.1 | 3.47 | 3.79 | 2.37 | 2.27 |
| 9 | 41 | 7.06 | 10.61 | 1.09 | 83.73 | 10.11 | 30.8 | 38.6 | 30.6 | 0.85 | 9.35 | 1073.1 | 4.68 | 139.92 | Extensive | 0 | Lowland | 474.1 | 3.32 | 3.23 | 3.12 | 3.22 |
| 10 | 42 | 7.41 | 10.24 | 7.24 | 108.36 | 11.74 | 32.2 | 39.8 | 28 | 0.90 | 9.12 | 1073.1 | 4.68 | 139.92 | Extensive | 0 | Lowland | 506.8 | 3.64 | 3.62 | 3.25 | 2.61 |
| 11 | 44 | 7.84 | 5.87 | 7.67 | 83.09 | 17.79 | 47.5 | 34.6 | 17.9 | 0.91 | 9.49 | 1066.47 | 4.64 | 138.84 | Extensive | 0 | Lowland | 461 | 3.27 | 3.14 | 3.14 | 3.08 |
| 12 | 47 | 7.55 | 7.82 | 8.07 | 87.98 | 12.40 | 36.3 | 40.5 | 23.2 | 0.88 | 9.57 | 1062.62 | 4.64 | 139.02 | Extensive | 0 | Lowland | 449.8 | 3.35 | 3.16 | 3.27 | 2.31 |
| 13 | 3 | 7.7 | 6.87 | 8.77 | 92.36 | 10.52 | 11.2 | 59.4 | 29.4 | 0.97 | 9.87 | 1023.14 | 4.64 | 138.3 | Low Intensive | 0 | Lowland | 389.7 | 3.42 | 3.29 | 3.18 | 3.08 |
| 14 | 13 | 7.75 | 8.66 | 2.28 | 54.78 | 20.08 | 31.4 | 38.1 | 30.5 | 0.85 | 9.11 | 1135.87 | 4.66 | 138.99 | Low Intensive | 0 | Lowland | 564.9 | 3.40 | 3.35 | 2.97 | 2.91 |
| 15 | 17 | 6.45 | 10.25 | 12.77 | 128.05 | 10.46 | 17.7 | 44.2 | 38.1 | 0.84 | 9.6 | 1112.17 | 4.61 | 136.61 | Low Intensive | 140.9 | Lowland | 422 | 3.38 | 3.04 | 3.65 | 3.05 |
| 16 | 21 | 7.67 | 5.00 | 8.78 | 99.59 | 24.98 | 58.6 | 23.2 | 18.2 | 1.00 | 9.74 | 942.81 | 4.84 | 137.22 | Low Intensive | 0 | Lowland | 397.1 | 3.22 | 2.98 | 3.22 | 2.76 |
| 17 | 23 | 8.03 | 6.56 | 7.50 | 107.71 | 22.42 | 31.3 | 52.1 | 16.6 | 0.93 | 9.49 | 993.97 | 4.8 | 138.93 | Low Intensive | 0 | Lowland | 439.4 | 3.50 | 3.22 | 3.29 | 2.89 |
| 18 | 26 | 6.92 | 13.94 | 4.42 | 90.21 | 10.70 | 19.8 | 30.8 | 49.4 | 0.71 | 9.02 | 990.62 | 4.79 | 137.8 | Low Intensive | 0 | Lowland | 536 | 3.43 | 3.07 | 3.42 | 3.30 |
| 19 | 27 | 7.39 | 10.15 | 5.01 | 96.64 | 10.70 | 30.7 | 38.4 | 30.9 | 0.83 | 8.74 | 1090.83 | 4.72 | 138.35 | Low Intensive | 0 | Lowland | 587.5 | 3.47 | 3.11 | 3.56 | 3.08 |
| 20 | 33 | 6.5 | 14.04 | 0.45 | 143.24 | 10.26 | 11.5 | 32.8 | 55.7 | 0.77 | 8.09 | 1195 | 4.62 | 137.8 | Low Intensive | 0 | Lowland | 740.8 | 3.33 | 3.07 | 3.06 | 2.95 |
| 21 | 36 | 5.81 | 6.38 | 1.90 | 45.44 | 10.93 | 27.1 | 49.6 | 23.3 | 1.03 | 9.15 | 1093.19 | 4.66 | 138.49 | Low Intensive | 0 | Lowland | 518.5 | 3.38 | 2.88 | 3.19 | 2.49 |
| 22 | 39 | 5.46 | 9.09 | 1.10 | 52.80 | 11.53 | 20.1 | 43.6 | 36.3 | 0.84 | 9.15 | 1106.13 | 4.67 | 139.46 | Low Intensive | 0 | Lowland | 532.9 | 3.38 | 2.07 | 3.35 | 3.07 |
| 23 | 43 | 6.3 | 6.29 | 1.61 | 77.03 | 10.23 | 41.7 | 41.2 | 17.1 | 1.09 | 9.41 | 1076.56 | 4.64 | 139.37 | Low Intensive | 0 | Lowland | 478.8 | 3.74 | 2.96 | 3.64 | 2.28 |
| 24 | 46 | 7.91 | 9.12 | 4.22 | 86.37 | 18.46 | 25.8 | 48.8 | 25.4 | 0.85 | 9.33 | 1083.45 | 4.65 | 138.76 | Low Intensive | 0 | Lowland | 475.2 | 3.20 | 3.15 | 3.34 | 2.89 |
| 25 | 1 | 7.53 | 10.31 | 6.34 | 112.39 | 14.13 | 19.1 | 50.9 | 30 | 0.82 | 9.86 | 1033.48 | 4.65 | 138.25 | Intensive | 109.9 | Lowland | 391.2 | 3.39 | 3.15 | 3.84 | 3.35 |
| 26 | 4 | 7.65 | 12.89 | 6.42 | 119.20 | 10.44 | 10.6 | 38 | 51.4 | 0.84 | 8.93 | 1113.26 | 4.73 | 138.89 | Intensive | 79 | Lowland | 491.7 | 3.50 | 3.08 | 3.93 | 3.38 |
| 27 | 8 | 6.71 | 9.28 | 5.88 | 102.83 | 10.97 | 24.2 | 34.6 | 41.2 | 0.87 | 9.29 | 1146.55 | 4.72 | 139.33 | Intensive | 242.8 | Lowland | 539.9 | 3.29 | 3.20 | 3.77 | 3.04 |
| 28 | 12 | 6.52 | 5.77 | 5.77 | 75.32 | 11.78 | 52.7 | 26.5 | 20.8 | 1.12 | 9.07 | 1135.87 | 4.66 | 138.99 | Intensive | 79 | Lowland | 571.7 | 3.16 | 3.07 | 4.00 | 3.24 |
| 29 | 19 | 6.59 | 8.88 | 3.72 | 88.10 | 9.65 | 30.6 | 40.4 | 29 | 0.90 | 9.25 | 954.39 | 4.81 | 137.97 | Intensive | 110.6 | Lowland | 484.4 | 3.32 | 3.13 | 3.99 | 3.34 |
| 30 | 22 | 7.95 | 7.06 | 13.00 | 128.09 | 21.21 | 28.9 | 55.9 | 15.2 | 0.94 | 9.47 | 993.97 | 4.8 | 138.93 | Intensive | 118.5 | Lowland | 442.8 | 3.49 | 3.16 | 3.94 | 3.04 |
| 31 | 25 | 6.9 | 9.32 | 2.95 | 79.86 | 9.57 | 36.6 | 36.8 | 26.6 | 0.82 | 9.1 | 990.62 | 4.79 | 137.8 | Intensive | 118.5 | Lowland | 524.4 | 3.75 | 3.08 | 3.99 | 2.70 |
| 32 | 30 | 7.39 | 8.67 | 8.32 | 87.47 | 16.57 | 28.7 | 40 | 31.3 | 0.96 | 8.93 | 1104.84 | 4.72 | 138.12 | Intensive | 58.2 | Lowland | 520.3 | 3.25 | 3.18 | 3.72 | 2.69 |
| 33 | 34 | 7.32 | 9.89 | 13.98 | 147.39 | 11.47 | 44.3 | 37.4 | 18.3 | 0.94 | 9.87 | 1089.7 | 4.7 | 138.43 | Intensive | 151.8 | Lowland | 391.3 | 3.58 | 3.14 | 4.00 | 3.09 |
| 34 | 35 | 7.35 | 10.47 | 28.03 | 181.98 | 10.08 | 25.3 | 46.1 | 28.6 | 0.91 | 9.5 | 1093.5 | 4.69 | 137.93 | Intensive | 103.0 | Lowland | 455.3 | 3.61 | 3.06 | 4.00 | 2.71 |
| 35 | 37 | 6.36 | 10.69 | 16.64 | 165.59 | 10.08 | 35.2 | 44.3 | 20.5 | 0.81 | 9.3 | 1208.83 | 4.74 | 138.08 | Intensive | 161.2 | Lowland | 549.5 | 3.20 | 3.05 | 4.00 | 2.91 |
| 36 | 38 | 6.09 | 8.65 | 4.61 | 116.01 | 9.65 | 27.1 | 49.4 | 23.5 | 0.94 | 9.34 | 1259.02 | 4.7 | 139.08 | Intensive | 64.8 | Lowland | 534.4 | 3.49 | 3.06 | 4.00 | 2.83 |
| 37 | 51 | 7.65 | 11.43 | 7.08 | 116.61 | 12.77 | 48.2 | 31.1 | 20.7 | 0.47 | 5.18 | 785.29 | 6.23 | 144.49 | Extensive | 0 | Subalpine | 1419.4 | 3.21 | 3.74 | 2.52 | 2.50 |
| 38 | 59 | 7.46 | 10.88 | 0.87 | 56.97 | 10.76 | 39.5 | 38.3 | 22.2 | 0.78 | 4.67 | 739.05 | 6.28 | 144.28 | Extensive | 0 | Subalpine | 1533.8 | 3.37 | 3.37 | 2.83 | 2.30 |
| 39 | 63 | 7.92 | 17.77 | 2.37 | 72.68 | 11.87 | 34.6 | 38.4 | 27 | 0.67 | 5.97 | 688.24 | 6.18 | 143.36 | Extensive | 0 | Subalpine | 1217 | 3.57 | 3.52 | 2.30 | 2.28 |
| 40 | 67 | 7.83 | 17.36 | 3.23 | 113.15 | 10.04 | 36.2 | 39.9 | 23.9 | 0.68 | 6.03 | 688.24 | 6.18 | 143.36 | Extensive | 0 | Subalpine | 1202.3 | 3.50 | 3.86 | 2.44 | 2.92 |
| 41 | 72 | 6.05 | 8.56 | 0.60 | 43.44 | 10.35 | 50.2 | 29.9 | 19.9 | 0.58 | 7.24 | 1628.17 | 5.65 | 143.62 | Extensive | 0 | Subalpine | 996.8 | 3.11 | 3.37 | 2.62 | 2.53 |
| 42 | 75 | 5.82 | 9.54 | 0.95 | 46.70 | 9.61 | 41.2 | 30.8 | 28 | 0.57 | 6.55 | 1671.97 | 5.69 | 144.42 | Extensive | 0 | Subalpine | 1145.3 | 3.18 | 3.48 | 2.81 | 2.50 |
| 43 | 81 | 5.26 | 9.65 | 0.58 | 29.87 | 8.94 | 25.7 | 42.7 | 31.6 | 0.47 | 6.47 | 1425.47 | 5.54 | 136.19 | Extensive | 0 | Subalpine | 1092.4 | 2.79 | 2.52 | 2.57 | 2.57 |
| 44 | 83 | 5.42 | 12.95 | 1.31 | 60.37 | 9.23 | 43.5 | 29 | 27.5 | 0.54 | 5.34 | 1687.18 | 5.64 | 143.3 | Extensive | 0 | Subalpine | 1389.5 | 2.61 | 2.94 | 2.94 | 2.90 |
| 45 | 86 | 5.13 | 6.43 | 0.90 | 42.83 | 14.81 | 47.5 | 46.4 | 6.1 | 0.52 | 5.29 | 1362.92 | 5.55 | 140.2 | Extensive | 0 | Subalpine | 1498.6 | 2.85 | 2.72 | 2.58 | 2.18 |
| 46 | 87 | 5.1 | 5.54 | 0.72 | 86.48 | 12.86 | 36.1 | 57.1 | 6.8 | 0.71 | 5.59 | 1200.58 | 5.48 | 137.93 | Extensive | 0 | Subalpine | 1379.4 | 2.72 | 3.09 | 3.01 | 2.88 |
| 47 | 91 | 5.34 | 8.34 | 1.61 | 103.28 | 11.98 | 48.5 | 42.8 | 8.7 | 0.66 | 5.5 | 1278.31 | 5.55 | 138.88 | Extensive | 0 | Subalpine | 1458.4 | 2.97 | 3.12 | 2.92 | 2.61 |
| 48 | 99 | 5.51 | 9.34 | 0.70 | 44.64 | 13.41 | 55.8 | 38.1 | 6.1 | 0.45 | 5.62 | 1198.92 | 5.41 | 138.5 | Extensive | 0 | Subalpine | 1338.7 | 2.77 | 3.31 | 3.57 | 2.06 |
| 49 | 50 | 7.59 | 17.88 | 1.47 | 79.17 | 8.76 | 41.6 | 32.4 | 26 | 0.68 | 5.06 | 785.29 | 6.23 | 144.49 | Low Intensive | 0 | Subalpine | 1445.1 | 3.57 | 3.63 | 2.48 | 3.08 |
| 50 | 54 | 7.55 | 15.87 | 1.94 | 68.89 | 9.12 | 32.3 | 37.1 | 30.6 | 0.69 | 4.74 | 739.05 | 6.28 | 144.28 | Low Intensive | 0 | Subalpine | 1518.4 | 3.54 | 3.34 | 3.07 | 3.08 |
| 51 | 62 | 7.74 | 13.43 | 6.14 | 115.41 | 13.73 | 43 | 37.8 | 19.2 | 0.75 | 5.74 | 688.24 | 6.18 | 143.36 | Low Intensive | 0 | Subalpine | 1269.3 | 3.37 | 3.11 | 3.42 | 2.97 |
| 52 | 66 | 7.66 | 11.41 | 8.64 | 109.37 | 11.03 | 44.3 | 33.9 | 21.8 | 0.84 | 5.77 | 688.24 | 6.18 | 143.36 | Low Intensive | 0 | Subalpine | 1263 | 3.28 | 3.52 | 3.05 | 2.61 |
| 53 | 68 | 6.04 | 7.07 | 1.36 | 56.86 | 9.55 | 36.2 | 39.7 | 24.1 | 0.69 | 6.5 | 1270.49 | 5.46 | 140.45 | Low Intensive | 0 | Subalpine | 1114.7 | 3.15 | 2.90 | 3.45 | 2.88 |
| 54 | 76 | 5.7 | 12.19 | 0.69 | 69.64 | 9.64 | 32.7 | 36.4 | 30.9 | 0.59 | 7.37 | 1363.87 | 5.71 | 139.28 | Low Intensive | 31.5 | Subalpine | 970.7 | 3.08 | 3.05 | 3.27 | 2.49 |
| 55 | 79 | 5.2 | 12.44 | 1.40 | 73.45 | 8.89 | 30.3 | 39.5 | 30.2 | 0.63 | 5.05 | 1380.46 | 5.57 | 140.24 | Low Intensive | 0 | Subalpine | 1483.6 | 2.84 | 3.04 | 3.29 | 3.20 |
| 56 | 80 | 5.76 | 12.10 | 1.60 | 115.88 | 7.68 | 16.1 | 45.1 | 38.8 | 0.68 | 7.04 | 1396.71 | 5.73 | 137.04 | Low Intensive | 0 | Subalpine | 1054.4 | 3.15 | 3.00 | 3.90 | 3.12 |
| 57 | 84 | 5.5 | 7.61 | 1.26 | 55.12 | 10.73 | 53.6 | 39.6 | 6.8 | 0.73 | 4.18 | 1286.47 | 5.59 | 139.43 | Low Intensive | 0 | Subalpine | 1695 | 2.72 | 3.29 | 3.48 | 3.15 |
| 58 | 89 | 4.96 | 8.09 | 1.02 | 59.74 | 11.58 | 40.4 | 51.2 | 8.4 | 0.55 | 5.14 | 1278.31 | 5.55 | 138.88 | Low Intensive | 0 | Subalpine | 1540 | 2.59 | 3.17 | 3.10 | 3.18 |
| 59 | 95 | 5.29 | 7.45 | 1.12 | 172.49 | 10.73 | 53.4 | 39.9 | 6.7 | 0.53 | 5.16 | 1217.6 | 5.48 | 139.12 | Low Intensive | 0 | Subalpine | 1484.9 | 2.86 | 3.28 | 3.66 | 2.94 |
| 60 | 96 | 5.36 | 7.27 | 2.94 | 51.27 | 11.69 | 56.3 | 37.1 | 6.6 | 0.66 | 5.14 | 1217.6 | 5.48 | 139.12 | Low Intensive | 0 | Subalpine | 1488.2 | 2.69 | 3.17 | 3.39 | 2.90 |
| 61 | 48 | 7.55 | 14.74 | 2.12 | 79.14 | 9.86 | 34.5 | 39.8 | 25.7 | 0.77 | 4.95 | 739.05 | 6.28 | 144.28 | Intensive | 23.7 | Subalpine | 1470.5 | 3.26 | 3.63 | 2.87 | 3.16 |
| 62 | 52 | 7 | 14.70 | 3.30 | 97.88 | 9.11 | 42.2 | 34.4 | 23.4 | 0.69 | 4.86 | 739.05 | 6.28 | 144.28 | Intensive | 18.8 | Subalpine | 1491.3 | 3.24 | 3.09 | 3.67 | 2.74 |
| 63 | 60 | 7.82 | 7.45 | 4.49 | 85.32 | 16.98 | 47.2 | 35.8 | 17 | 0.87 | 5.47 | 688.24 | 6.18 | 143.36 | Intensive | 11.8 | Subalpine | 1331.6 | 3.56 | 3.37 | 3.35 | 2.96 |
| 64 | 64 | 7.5 | 12.41 | 7.33 | 125.58 | 10.08 | 45.5 | 31.6 | 22.9 | 0.81 | 5.93 | 688.24 | 6.18 | 143.36 | Intensive | 45.1 | Subalpine | 1226.5 | 3.34 | 3.13 | 3.72 | 3.18 |
| 65 | 70 | 5.95 | 7.69 | 0.77 | 68.38 | 8.95 | 29.7 | 44 | 26.3 | 0.85 | 7.67 | 1380.46 | 5.57 | 140.24 | Intensive | 49.6 | Subalpine | 899.3 | 3.26 | 3.00 | 3.84 | 3.00 |
| 66 | 71 | 7.3 | 15.72 | 2.05 | 111.37 | 9.08 | 35.8 | 42.2 | 22 | 0.65 | 7.6 | 1628.17 | 5.65 | 143.62 | Intensive | 45.4 | Subalpine | 921.1 | 3.16 | 3.05 | 3.54 | 2.74 |
| 67 | 74 | 6.34 | 14.10 | 0.92 | 112.71 | 9.17 | 47.1 | 30.4 | 22.5 | 0.63 | 7.71 | 1628.17 | 5.65 | 143.62 | Intensive | 46.2 | Subalpine | 898 | 3.41 | 3.04 | 3.71 | 3.24 |
| 68 | 77 | 5.51 | 12.57 | 1.05 | 102.94 | 8.38 | 29.1 | 37.5 | 33.4 | 0.70 | 7.48 | 1374.08 | 5.77 | 137.95 | Intensive | 111.8 | Subalpine | 947.3 | 3.12 | 2.99 | 3.83 | 3.12 |
| 69 | 88 | 5.68 | 6.69 | 1.42 | 115.78 | 11.22 | 34.5 | 57.3 | 8.2 | 0.82 | 5.52 | 1200.58 | 5.48 | 137.93 | Intensive | 63.0 | Subalpine | 1396.1 | 3.03 | 3.02 | 4.04 | 3.30 |
| 70 | 92 | 5.23 | 7.92 | 1.41 | 129.00 | 11.21 | 43.7 | 46.5 | 9.8 | 0.72 | 5.86 | 1278.31 | 5.55 | 138.88 | Intensive | 88.24 | Subalpine | 1376.9 | 2.70 | 2.98 | 4.03 | 3.20 |
| 71 | 93 | 5.48 | 7.12 | 2.30 | 100.20 | 11.86 | 58.3 | 35.8 | 5.9 | 0.65 | 5.5 | 1198.92 | 5.41 | 138.5 | Intensive | 93.23 | Subalpine | 1366.5 | 2.86 | 2.95 | 3.61 | 3.09 |
| 72 | 97 | 5.29 | 5.70 | 0.74 | 87.95 | 11.25 | 52.1 | 40.8 | 7.1 | 0.77 | 5.4 | 1198.92 | 5.41 | 138.5 | Intensive | 35.2 | Subalpine | 1388.6 | 2.93 | 3.06 | 3.42 | 3.36 |
|  |  |  |  |  |  |  |  |  |  |  |  |  |  |  |  |  |  |  |  |  |  |  |

^a^ The number refers to the amplicon sequences (BIOCHxxE) deposited for the respective sites at the NCBI SRA archive (accession number PRJNA641340)

**Supplementary Table 2**: Summary statistics of the best-fitting generalized linear models (GLMs) predicting presence/absence (binary response) of individual fungal ASVs based on environmental predictors. each row presents the model estimates for the intercept and significant predictors, along with their standard error, z-value, p-value, and 95 % confidence interval (CI). Model fit is assessed using the Akaike Information Criterion (AIC) and Area Under the Receiver Operating Characteristic Curve (AUC). AIC is a measure of model quality that balances model fit and complexity. AUC evaluates the model’s ability to distinguish between presence and absence; values range from 0.5 (no discrimination) to 1 (perfect discrimination).

|  | **Predictors** | **estimate** | **Std. error** | **z-value** | **p-value** | **CI (95 %)** | **AIC** | **AUC** |
| --- | --- | --- | --- | --- | --- | --- | --- | --- |
| **ASV 3** | Intercept | -10.47 | 2.35 | -4.46 | 8.29e-06 | -15.55 / -6.22 |  |  |
|  | pH | 1.65 | 0.36 | 4.63 | 3.62e-06 | 1.01 / 2.43 |  |  |
|  |  |  |  |  |  |  | 68.43 | 0.85 |
| **ASV 15** | Intercept | -1.58 | 3.09 | -0.51 | 0.608 | -7.73 / 4.53 |  |  |
|  | pH | -0.77 | 0.39 | -1.97 | 0.048 | -1.58 / -0.03 |  |  |
|  | Avail. Phosphorus | -0.15 | 0.08 | -1.88 | 0.061 | -0.33 / -0.01 |  |  |
|  | Temperature | 2.35 | 1.28 | 1.84 | 0.067 | -0.05 / 5.03 |  |  |
|  |  |  |  |  |  |  | 93.67 | 0.73 |
| **ASV 22** | Intercept | -6.34 | 1.92 | -3.29 | 9.87e-04 | -10.52 / -2.90 |  |  |
|  | Total Phosphorus | 0.02 | 0.01 | 2.28 | 0.023 | 0.00 / 0.04 |  |  |
|  | Bulk Soil Density | 5.25 | 2.03 | 2.58 | 9.80e-03 | 1.49 / 9.55 |  |  |
|  |  |  |  |  |  |  | 89.50 | 0.76 |
| **ASV 82** | Intercept | 137.9 | 52.91 | 2.61 | 9.15e-03 | 54.67 / 259.01 |  |  |
|  | Bulk Soil Density | 8.72 | 2.84 | 3.07 | 2.12e-03 | 3.74 / 15.09 |  |  |
|  | Solar Radiation | -1.04 | 0.39 | -2.70 | 6.86e-03 | -1.93 / -0.44 |  |  |
|  |  |  |  |  |  |  | 63.08 | 0.89 |
| **ASV 346** | Intercept | 24.28 | 15.97 | 1.52 | 0.128 | 5.98 / 73.52 |  |  |
|  | Mean Sunshine Hours | -5.39 | 3.39 | -1.59 | 0.111 | -15.89 / -1.57 |  |  |
|  |  |  |  |  |  |  | 44.64 | 0.81 |
| **ASV 464** | Intercept | 16.05 | 5.98 | 2.69 | 7.23 e-03 | 6.62 / 30.38 |  |  |
|  | Temperature | -1.82 | 0.79 | -2.30 | 0.021 | -3.76 / -0.61 |  |  |
|  | Bulk Soil Density | -10.60 | 4.76 | -2.23 | 0.026 | -21.50 / -2.25 |  |  |
|  |  |  |  |  |  |  | 35.01 | 0.96 |

|  | **Predictors** | **Degrees of freedom** | **Sum of Squares** | **R^2^** | **F-statistic** | **p-value** |
| --- | --- | --- | --- | --- | --- | --- |
| Plant community | Geographic Region | 1 | 2.38 | 0.10 | 7.85 | 0.001 |
|  | Residual | 70 | 21.25 | 0.90 |  |  |
|  |  |  |  |  |  |  |
| Plant community | Management | 2 | 4.20 | 0.18 | 7.45 | 0.001 |
|  | Residual | 69 | 19.44 | 0.82 |  |  |
|  |  |  |  |  |  |  |
| Plant community | pH | 1 | 2.18 | 0.09 | 9.63 | 0.001 |
|  | Total Soil Organic Matter | 1 | 0.63 | 0.03 | 2.79 | 0.003 |
|  | Available Phosphorus | 1 | 1.33 | 0.06 | 5.88 | 0.001 |
|  | Total Phosphorus | 1 | 0.57 | 0.02 | 2.53 | 0.003 |
|  | Carbon to Nitrogen Ratio | 1 | 0.47 | 0.02 | 2.09 | 0.013 |
|  | Sand | 1 | 0.71 | 0.03 | 3.16 | 0.002 |
|  | Silt | 1 | 0.49 | 0.02 | 2.15 | 0.014 |
|  | Bulk Soil Density | 1 | 0.80 | 0.03 | 3.56 | 0.001 |
|  | Temperature | 1 | 0.48 | 0.02 | 2.12 | 0.018 |
|  | Precipitation | 1 | 0.33 | 0.01 | 1.47 | 0.107 |
|  | Mean Sunshine Hours | 1 | 0.60 | 0.03 | 2.67 | 0.006 |
|  | Solar Radiation | 1 | 0.37 | 0.02 | 1.64 | 0.054 |
|  | Total Applied Nitrogen | 1 | 1.27 | 0.05 | 5.63 | 0.001 |
|  | Residual | 58 | 13.28 | 0.57 |  |  |
|  |  |  |  |  |  |  |
| Plant community | ASV 3 | 1 | 0.97 | 0.04 | 3.23 | 0.002 |
|  | ASV 15 | 1 | 0.56 | 0.02 | 1.86 | 0.017 |
|  | ASV 22 | 1 | 0.90 | 0.04 | 3.00 | 0.001 |
|  | ASV 82 | 1 | 0.97 | 0.04 | 3.23 | 0.002 |
|  | ASV 346 | 1 | 0.23 | 0.01 | 0.75 | 0.798 |
|  | ASV 464 | 1 | 0.47 | 0.02 | 1.57 | 0.032 |
|  | Residual | 65 | 19.54 | 0.83 |  |  |

**Supplementary Table 3:** Plant communities correlate to geographic region (lowland or subalpine), management intensity and environmental factors. Bray-Curtis dissimilarity of the plant community was correlated separately with lowland and subalpine as well as management intensity, and was correlated with a full model with all environmental factors and management factors, as well as with a full model with all six *Metarhizium* and *Beauveria* ASVs, using 999 permutations.

**Supplementary Table 4:** Indicator plant species for fungal ASVs. Indicator species analysis and Spearman correlation were performed for four Metarhizium and two Beauveria ASVs with plant species occurring at more than 10 sites. Indicator values were calculated using presence/absence data of fungal ASVs and either presence/absence of or percent area covered by a plant species per site. Spearman's rho was calculated based on the relative sequence abundance of fungal ASVs and plant species coverage. P-values were adjusted for multiple comparisons using the Benjamini-Hochberg correction. Significant indicator values (>0.5) and significant correlations (>0.3) are highlighted in yellow. Plant species that were significant and above the threshold in all three analyses are shown in **bold** and listed in Table 2.

|  |  |  | Fungal presence/absence  Plant presence/absence | | Fungal presence/absence  Plant coverage | | Fungal abundance  Plant coverage | | |
| --- | --- | --- | --- | --- | --- | --- | --- | --- | --- |
| **Fungal ASV** | **Plant** |  | **Indicator value** | **p-value** | **Indicator value** | **p-value** | | **Rho** | **p-value** |
| ASV 3 | ***Galium mollugo*** | | **0.66** | **0.001** | **0.85** | **0.001** | | **0.66** | **3.04e-08** |
|  | ***Achillea millefolium*** | | **0.55** | **0.002** | **0.84** | **0.001** | | **0.45** | **5.79e-04** |
|  | ***Arrhenatherum elatius*** | | **0.53** | **0.004** | **0.82** | **0.002** | | **0.49** | **2.62e-04** |
|  | ***Helictotrichon pubescens*** | | **0.50** | **0.001** | **0.73** | **0.001** | | **0.51** | **1.40e-04** |
|  | *Salvia pratensis* | | 0.46 | 0.001 | 0.69 | 0.002 | | 0.44 | 8.39e-04 |
|  | *Knautia arvensis* | | 0.35 | 0.026 | 0.67 | 0.005 | | 0.41 | 1.58e-03 |
|  | *Ranunculus bulbosus* | | 0.42 | 0.001 | 0.65 | 0.002 | | 0.38 | 4.34e-03 |
|  | *Bromus erectus* | | 0.43 | 0.002 | 0.63 | 0.013 | | 0.42 | 1.58e-03 |
|  | *Veronica arvensis* | | 0.30 | 0.032 | 0.62 | 0.012 | | 0.37 | 5.47e-03 |
|  | *Lotus corniculatus* | | 0.49 | 0.006 | - | - | | 0.43 | 9.57e-04 |
|  | *Plantago media* | | 0.35 | 0.003 | 0.61 | 0.005 | | 0.40 | 2.41e-03 |
|  | *Sanguisorba minor* | | 0.28 | 0.050 | 0.58 | 0.013 | | 0.30 | 2.57e-02 |
|  | *Medicago lupulina* | | 0.39 | 0.003 | 0.58 | 0.041 | | 0.41 | 1.74e-03 |
|  | *Veronica persica* | | 0.27 | 0.003 | 0.52 | 0.008 | | 0.36 | 6.13e-03 |
|  | *Picris hieracioides* | | 0.26 | 0.019 | 0.54 | 0.004 | | 0.30 | 2.59e-02 |
|  | *Thymus serpyllum* | | 0.19 | 0.042 | 0.47 | 0.036 | | 0.14 | 3.49e-01 |
|  | *Glechoma hederacea* | | 0.31 | 0.075 | 0.61 | 0.033 | | 0.29 | 3.27e-02 |
|  | *Centaurea scabiosa* | | 0.28 | 0.018 | 0.57 | 0.013 | | 0.20 | 1.71e-01 |
|  | *Plantago lanceolata* | | 0.63 | 0.001 | - | - | | 0.32 | 1.29e-02 |
|  | *Centaurea jacea* | | 0.37 | 0.007 | - | - | | 0.41 | 1.58e-03 |
|  | *Rumex acetosa* | | 0.31 | 0.093 | - | - | | 0.37 | 4.35e-03 |
|  | *Prunella vulgaris* | | 0.33 | 0.251 | - | - | | 0.34 | 1.05e-02 |
| ASV 15 | *Heracleum sphondylium* | | 0.42 | 0.005 | 0.69 | 0.003 | | 0.36 | 1.64e-02 |
|  | *Anthriscus sylvestris* | | 0.22 | 0.044 | 0.52 | 0.015 | | 0.39 | 3.38e-02 |
|  | *Cardamine pratensis* | | 0.28 | 0.027 | 0.57 | 0.006 | | 0.29 | 1.86e-01 |
| ASV 22 | *Lolium multiflorum* | | 0.36 | 0.009 | 0.66 | 0.002 | | 0.41 | 6.80e-03 |
|  | *Lolium perenne* | | 0.51 | 0.003 | - | - | | 0.40 | 6.80e-03 |
|  | *Bellis perennis* | | 0.29 | 0.095 | 0.60 | 0.041 | | 0.24 | 1.31e-01 |
|  | *Taraxacum officinale* | | 0.45 | 0.274 | - | - | | 0.40 | 6.80e-03 |
| ASV 82 | ***Poa trivialis*** |  | **0.51** | **0.003** | **0.76** | **0.002** | | **0.47** | **7.02e-04** |
|  | *Holcus lanatus* | | 0.46 | 0.005 | 0.73 | 0.001 | | 0.33 | 2.53e-02 |
|  | *Anthoxanthum odoratum* | | 0.27 | 0.020 | 0.66 | 0.002 | | 0.41 | 4.83e-03 |
|  | *elymus repens* | | 0.34 | 0.001 | 0.56 | 0.002 | | 0.45 | 1.18e-03 |
|  | *Lolium multiflorum* | | 0.31 | 0.037 | 0.58 | 0.036 | | 0.33 | 2.53e-02 |
|  | *Alopecurus pratensis* | | 0.27 | 0.009 | 0.58 | 0.003 | | 0.33 | 2.53e-02 |
|  | *Poa pratensis* |  | 0.56 | 0.002 | - | - | | 0.49 | 7.02e-04 |
|  | *Centaurea jacea* | | 0.32 | 0.021 | 0.58 | 0.022 | | 0.26 | 7.20e-02 |
|  | *Veronica arvensis* | | 0.31 | 0.032 | 0.58 | 0.034 | | 0.27 | 7.20e-02 |
|  | *Medicago lupulina* | | 0.29 | 0.079 | 0.60 | 0.016 | | 0.22 | 1.37e-01 |
|  | *Glechoma hederacea* | | 0.31 | 0.090 | 0.61 | 0.022 | | 0.22 | 1.37e-01 |
|  | *Agrostis stolonifera* | | 0.24 | 0.033 | - | - | | 0.32 | 3.39e-02 |
|  | *Ranunculus acris* | | 0.51 | 0.012 | - | - | | 0.19 | 1.81e-01 |
| ASV 346 | ***Medicago lupulina*** | | **0.58** | **0.007** | **0.80** | **0.001** | | **0.39** | **8.77e-03** |
|  | ***Knautia arvensis*** | | **0.56** | **0.008** | **0.78** | **0.003** | | **0.38** | **1.03e-02** |
|  | ***Ajuga reptans*** | | **0.60** | **0.008** | **0.88** | **0.002** | | **0.38** | **1.03e-02** |
|  | ***Prunella vulgaris*** | | **0.61** | **0.010** | **0.84** | **0.003** | | **0.38** | **1.03e-02** |
|  | ***Arrhenatherum elatius*** | | **0.66** | **0.005** | **0.84** | **0.002** | | **0.34** | **2.49e-02** |
|  | ***Bromus erectus*** | | **0.67** | **0.002** | **0.82** | **0.002** | | **0.40** | **7.99e-03** |
|  | ***Centaurea jacea*** | | **0.60** | **0.003** | **0.81** | **0.001** | | **0.48** | **8.01e-04** |
|  | ***Galium mollugo*** | | **0.59** | **0.019** | **0.79** | **0.011** | | **0.34** | **2.24e-02** |
|  | ***Sanguisorba minor*** | | **0.60** | **0.005** | **0.78** | **0.003** | | **0.41** | **7.99e-03** |
|  | ***Holcus lanatus*** | | **0.74** | **0.001** | **0.75** | **0.013** | | **0.36** | **1.36e-02** |
|  | *Rumex acetosa* | | 0.44 | 0.084 | 0.74 | 0.015 | | 0.25 | 1.39e-01 |
|  | *Glechoma hederacea* | | 0.45 | 0.042 | 0.74 | 0.015 | | 0.25 | 1.39e-01 |
|  | *Salvia pratensis* | | 0.46 | 0.048 | 0.68 | 0.026 | | 0.22 | 1.97e-01 |
|  | *Helictotrichon pubescens* | | 0.44 | 0.054 | 0.68 | 0.035 | | 0.24 | 1.49e-01 |
|  | *Plantago media* | | 0.46 | 0.048 | 0.62 | 0.041 | | 0.24 | 1.47e-01 |
|  | *Primula veris* | | 0.35 | 0.035 | 0.61 | 0.012 | | 0.30 | 6.22e-02 |
|  | *Festuca pratensis* | | 0.52 | 0.028 | - | - | | 0.26 | 1.39e-01 |
|  | *Lotus corniculatus* | | 0.55 | 0.051 | 0.88 | 0.002 | | 0.33 | 2.85 e-02 |
| ASV 464 | ***Silene vulgaris*** | | **0.70** | **0.001** | **0.88** | **0.001** | | **0.51** | **2.87e-04** |
|  | ***Festuca rubra*** |  | **0.60** | **0.044** | **0.86** | **0.001** | | **0.44** | **1.55e-03** |
|  | ***Cynosurus cristatus*** | | **0.55** | **0.004** | **0.74** | **0.006** | | **0.44** | **1.55e-03** |
|  | ***Chaerophyllum hirsutum*** | | **0.52** | **0.001** | **0.73** | **0.001** | | **0.50** | **3.35e-04** |
|  | ***Agrostis capillaris*** | | **0.53** | **0.004** | **0.76** | **0.001** | | **0.43** | **1.85e-03** |
|  | *Rhinanthus alectorolophus* | | 0.48 | 0.027 | 0.80 | 0.001 | | 0.36 | 1.16e-02 |
|  | *Campanula scheuchzeri* | | 0.49 | 0.006 | 0.75 | 0.001 | | 0.46 | 9.00e-04 |
|  | *Leontodon hispidus* | | 0.45 | 0.011 | 0.75 | 0.001 | | 0.41 | 2.74e-03 |
|  | *Briza media* |  | 0.46 | 0.008 | 0.69 | 0.005 | | 0.34 | 2.07e-02 |
|  | *Thymus serpyllum* | | 0.42 | 0.007 | 0.69 | 0.001 | | 0.41 | 2.74e-03 |
|  | *Leucanthemum vulgare* | | 0.42 | 0.069 | 0.75 | 0.027 | | 0.26 | 7.49e-02 |
|  | *Silene dioica* |  | 0.41 | 0.016 | 0.65 | 0.004 | | 0.35 | 1.66e-02 |
|  | *Geranium sylvaticum* | | 0.34 | 0.104 | 0.60 | 0.047 | | 0.23 | 1.23e-01 |
|  | *Hypericum maculatum* | | 0.31 | 0.041 | 0.58 | 0.023 | | 0.30 | 4.49e-02 |
|  | *Pimpinella major* | | 0.27 | 0.151 | 0.58 | 0.035 | | 0.20 | 1.87e-01 |
|  | *Carex caryophyllea* | | 0.39 | 0.017 | 0.54 | 0.036 | | 0.29 | 5.48e-02 |
